# Gratefully Received, Gratefully Repaid: The Role of Perceived Fairness in Cooperative Interactions

**DOI:** 10.1371/journal.pone.0114976

**Published:** 2014-12-08

**Authors:** Lawrence K. Ma, Richard J. Tunney, Eamonn Ferguson

**Affiliations:** 1 Personality, Social Psychology, and Health Research Group, School of Psychology, University of Nottingham, Nottingham, United Kingdom; 2 School of Psychology, University of Nottingham, Nottingham, United Kingdom; University of Pennsylvania, United States of America

## Abstract

It is well documented that people would remunerate fair behaviours and penalize unfair behaviours. It is argued that individuals’ reactions following the receipt of a gift depend on the perceived intentions of the donors. *Fair* intentions should prompt positive affect, like gratitude, triggering cooperative behaviours; while intended unfairness should trigger negative affect, like anger, fostering anti-social actions. It is, however, contended that when people lack information to infer others’ intention they may use ‘normative’ beliefs about fairness - what a typical fair individual ‘should’ do in these circumstances – to guide their behaviour. In this experiment we examined this assertion. We had 122 participants play a one-shot, double-anonymous game with half playing as potential helpers (P1s) and half as recipients (P2s). Whether a participant was a P1 or P2 was chance-determined and all participants knew this. P1s decided whether to help P2s and whether to make their help unconditional (no repayment needed) or conditional (full or ‘taxed’ repayment). P2s decided whether to accept the offer and whatever conditions attached but were blind to the list of helping options available to P1s. We anticipated that recipients would refer to the ‘injunctive norm’ that ‘fair people should help “for free” when it is only by chance that they are in a position to help’. Therefore, without knowing P1s’ different helping options, unconditional offers should be rated by recipients as fairer than conditional offers, and this should be linked to greater gratitude with greater gratitude linked to greater reciprocation. Path analyses confirmed this serial mediation. The results showed that recipients of unconditional offers, compared to conditional ones, interpreted the helpers’ motives as more helpful, experienced greater gratitude and were more eager to reciprocate. The behavioural data further revealed that, when given a latter option to default, 38% of recipients of conditional offers did so.

## Introduction

The ‘reciprocity rule’ stipulates that ‘we should attempt to repay, in kind, what another person has provided us (pp.20)’ [1]. Fehr and colleagues [2]–[4] further make a distinction between the tendency to remunerate (or be cooperative with) fair behaviours (positive reciprocity), and the tendency to punish (or be hostile towards) acts we perceive as unkind or unfair (negative reciprocity). Positive reciprocity is often mediated by the perceived trustworthiness of the donor [5], [6] and negative reciprocity via negative emotions such as anger directed towards the transgressor [7], [8].

The above suggests that when an individual decides whether to penalize or reward a partner the attribution of the partners’ intentions is crucial [9]. Indeed Falk and Fischbacher [10] argue that reciprocation (or retaliation) is dependent upon the recipient’s evaluation of the donor’s intention. As such, reciprocity constitutes more than a recipient’s knee-jerk reaction, as the beneficiary also ponders why he/she was offered what they were.

Greenberg and Westcott [11] argued that a pro-social gesture which implies good intentions on behalf of the donor should elicit positive emotions such as gratitude in the recipient towards the donor [12]–[15], and these positive emotions can motivate reciprocation [16]–[19]. Indeed, Nowak and Roch [20] have argued that gratitude is a key emotion to sustain both direct and indirect reciprocation. The corollary of this is that unfair acts that are perceived to reflect malicious intentions should elicit negative emotions such as indebtedness and anger [21], [22]. Indebtedness is an aversive psychological state of tension [23] that is associated with action tendencies [24] like inhibition (e.g. to ‘feel like shutting myself off from my surroundings’) and avoidance (e.g. ‘to feel like ignoring my friend’ (pp.229)) [25].

While people attend to others’ intentions they ‘do not attribute good or bad intention in a vacuum (pp.112)’, as Bicchieri [26] contended ‘an intention is only good or bad against background of expectations (pp.112)’. These expectations constitute, amongst other things, the ‘injunctive norm’ about what one ‘ought to do’ in a given situation [27]–[29]. Indeed, the idea that injunctive norms are the basis of fairness judgment forms the basis of many models of social cooperation [30]–[33].

In the present paper we endeavoured to examine this idea using a one-shot variant of the trust game. Particularly, in this game 1) all players were anonymous to one another and the experimenters (thereby avoiding any incentives to build reputations); 2) players interacted in dyads with one being a potential helper (P1 hereafter) and the other playing a potential recipient (P2 hereafter), 3) who ended up as either P1 or P2 in every pair, was by pure chance thus removing any attributions about ability; and 4) P2s were completely unknowledgeable about the options P1s had available to them. P1s could offer unconditional help (ask for no repayment, and P2s could not repay) or conditional help (full repayment of repayment with tax). Thus, under these conditions, P2s could only rely on their beliefs of what people ‘ought’ to do in this context – their injunctive norm [34] – as the basis of judging how fair (or unfair) P1 was with respect to the offer they made.

Thus we examined whether the difference in the types of helping offered (unconditional vs. conditional) would be sufficient to induce different attributions of fairness in recipients towards the helper. The null hypothesis would state that in the absence of any information about the options open to the helpers, all help, conditional or otherwise, would be perceived as equally fair. Alternatively, it is plausible to hypothesize that individuals make use injunctive ‘norms’ (i.e. how people ought to behave) of fairness. When somebody’s good fortune is acquired by chance, rather than the owner having to earn the ‘Property Rights’ [33], [35] or the ‘Entitlement’ [31], [36] to that money, then in general ‘fair’ individuals should help others and help unconditionally. This hypothesis is supported by evidence in social psychology, social cooperation and behavioural economics [37]–[40]. As such, we anticipated a difference in attributions of helpers’ intentions to be fair, even in the absence of any knowledge about the choices facing the donor, purely on the basis of whether the offer is conditional or not. That is, regardless of not knowing the options available (to their helpers), P2s will make use of an injunctive normative belief that fair people will help for free when that help is easy to implement and when the helpers’ potency to help was ‘assigned’ (via chance) instead of ‘earned’.

As well as hypothesizing that an unconditional offer would trigger a favourable appraisal of the donor’s intentions, compared to a conditional one; we also believed that this would generate a greater level of gratitude [41], [42]. We further expect that this gratitude would translate to an increased willingness for the recipients to reciprocate in the future [43], [44]. A conditional offer, on the contrary, would not only elicit a more unfavourable evaluation of the donors’ intent but also reduced desire to reciprocate [41], with this related to one’s decisions to default on his/her agreement (e.g., not returning the donation) [45]. Thus we test a serial multiple mediation model [46] which explores if the effect of offer conditionality (unconditional vs. conditional) on reciprocity tendency is mediated serially by two psychological processes: (1) initial attributions that the donor genuinely intend to be helpful and (2) feelings of gratitude.

## Methods

### Participants

One hundred and twenty-two students from University of Nottingham participated in the experiment (72 were females, mean age: 21.8 years, SD = 3.7 years). Each participant was paid an inconvenience allowance of £2 (US$ = 3.27) and was given an opportunity to earn up to £3.50 (US$ = 5.72) more.

### Game and its Rationale

The experiment was a modified trust game conducted using Z-Tree [47]. To begin with each participant was given a fair die to roll and was seated in a separate cubicle. Experimental session took place with groups of 4 to 10 participant, with participants in each session randomly assigned to play in dyads, with one participant being the potential donor (P1) and the other playing a potential recipient (P2).

Each player was initially endowed with 150 money-equivalent ‘Bonus Points’ and was told that he/she needed *at least 50 points more* to reach the ‘Bonus Threshold’ (i.e. 200) to claim extra money (i.e. the bonus). We allocated the initial endowments this way to create a situation in which all players were, to begin with, equal in the sense that they were all entitled to no additional bonuses as their initial bonus points stood at 150. It was made clear to every participant that each bonus point would be worth one penny (US$ = 0.17 cents) but only when he/she made the 200 point threshold was he/she entitled to any bonus. The only way to reach that threshold, as we told our participants, was by rolling a fair, six-sided die once. The possible payoffs were a loss of –50 points, 0 points, and gains of 50, 100, 150, and 200 points depending on the number on the dice.

However, it was emphasized that a larger number on the dice did not guarantee a more favourable allocation of bonus points. The purpose of this die-rolling arrangement was to lead the participants to believe that what segregated the ‘winners’ (i.e. hitting the threshold) from the ‘losers’ (i.e. missing the threshold) was better luck instead of ability. Every participant then learned that in this game he/she had a ‘partner’, who was unidentifiable and anonymous. They were reminded that ‘neither joint effort nor competition with your partner is needed’.

Nevertheless, the participants’ scores were pre-determined. Regardless of the actual die-roll, P1s always received a ‘Die-rolling’ score 200 (thus they ended up with 350 points: their initial 150 + 200) that exceeded the 200 point threshold, while P2s would always fall short of the 200 point threshold. Half the P2s (N = 30) ended up with a final total of 100 bonus point (that is their initial 150 minus 50 points form the dice roll) and the other P2s (N = 31) ended up with 150 points (that is their initial 150 plus zero from the dice roll). This difference between the P2s was aimed to manipulate the potential cost of helping for the P1s. The P2s with 100 point were in the ‘High-Cost’ helping condition, and would require twice the magnitude of their partners’ (or potential helpers’) minimum ‘donations’ (i.e. 100) compared to the ‘Low-Cost’ recipients with a 150 points (i.e. 50), to reach the 200 point threshold. Each player then learned about his/her payoff and that of his/her partner.

At this point both P1s’ and P2s’ provided attributions regarding their partners’ Die-rolling scores. Specifically they indicated if they thought that their partners’ scores were due to (1) Luck (‘To what extent you think that your partner’s High (Low) score is attributable to his/her *good (bad) luck*), and (2) Ability (‘To what extent you think that your partner’s High (Low) score is because of his/her *capability (incompetence)*’). Both items were measured using 7-point Likert type scales (1 = ‘Not At All’ and 7 = ‘Completely’). In so doing we checked if our participants perceived their partners’ (good or bad) outcomes as chance-determined rather than ‘earned’.

### ‘Free’ versus ‘Charged’ Offers

**Figure 1:**
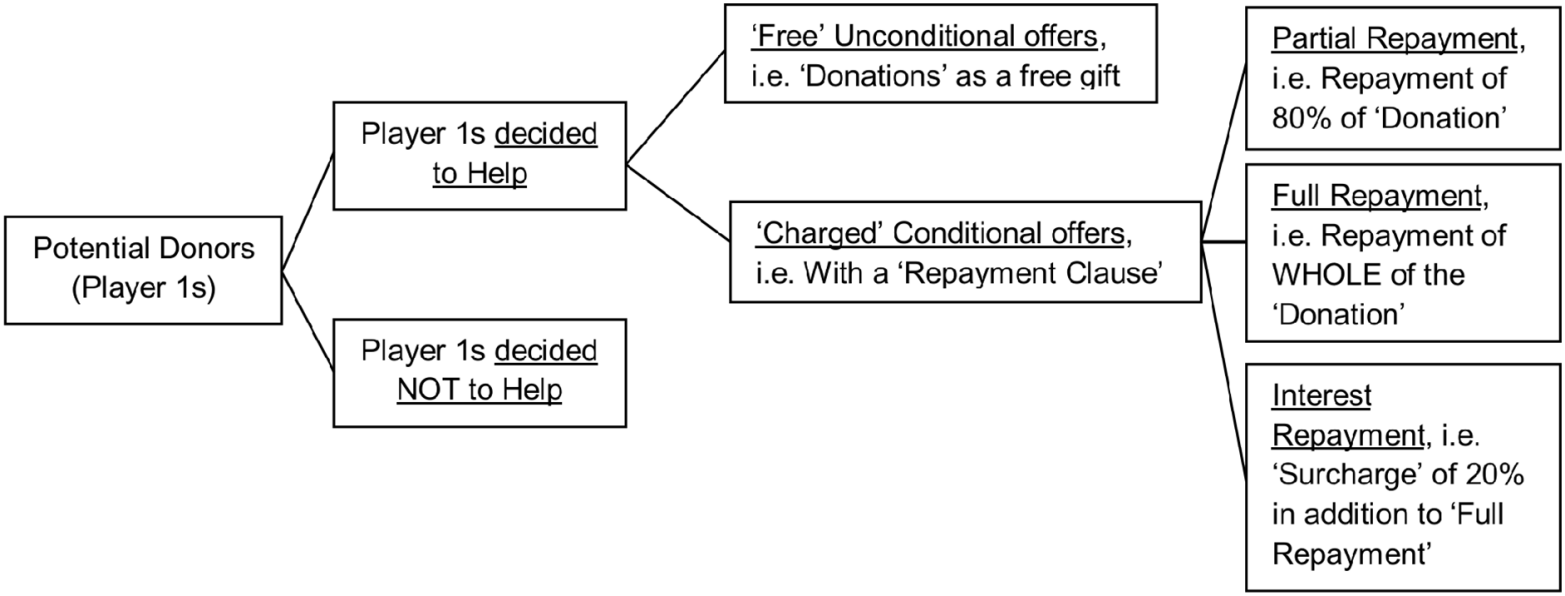
The flow of decisions made by a potential helper (Player 1).

Helper’s Decisions. P1s, who were better off financially than P2s, were then asked if they would help P2s by transferring part of their excess bonus points (i.e. 350 – 200 ‘Bonus Threshold’ = 150 points). Donors could help either *unconditionally* (i.e. to give away the points as a gift and expect no repayment: repayment free offer), or *conditionally* by applying a ‘repayment clause’ for the amount donated. If a P1 chose to offer conditional help, he/she was asked to pick a level of repayment as the ‘repayment clause’ for his/her recipient (Fig. 1). All these decisions were made privately and P2s were totally blind to the list of choices that were available to P1s.

#### Recipients’ Reactions

After P1s made the decisions their P2 partners were shown, firstly, whether P1s decided to help; and if so, whether the offers were conditional or unconditional and if they were conditional what the repayment requirements were (P2s were never aware that P1s had the choice to make offers either conditional or unconditional, they just saw the offer that P1 decided on). P2s who received an offer were then asked to decide whether to accept it or not. It was made clear to them that the P2s would always be financially better off by any offer of help compared to no offer of help. We then assessed recipients’ perception of their donors decisions using the following constructs: (1) State Annoyance (‘I am annoyed by my partner’s decision.’), (2) State Gratitude (Average of items ‘I am thankful for my partner’s decision’ and ‘I feel grateful for what my partner does for me’, Cronbach’s α = 0.931), (3) State Indebtedness (‘I feel indebted to my partner’), (4) Obligation (‘I feel obliged to repay my partner’), (5) Perceived Genuine Helpfulness (‘My partner's transfer is motivated by his/her sincere desire to help me’), (6) Perceived Reasonableness (‘My partner’s decision is reasonable’), and (7) Reciprocating Tendency (‘I am eager to help my partner out if he/she is in need in the near future’). All were measured using 7-point Likert type scales (where 1 = ‘Not At All’ and 7 = ‘Completely’).

**Figure 2:**
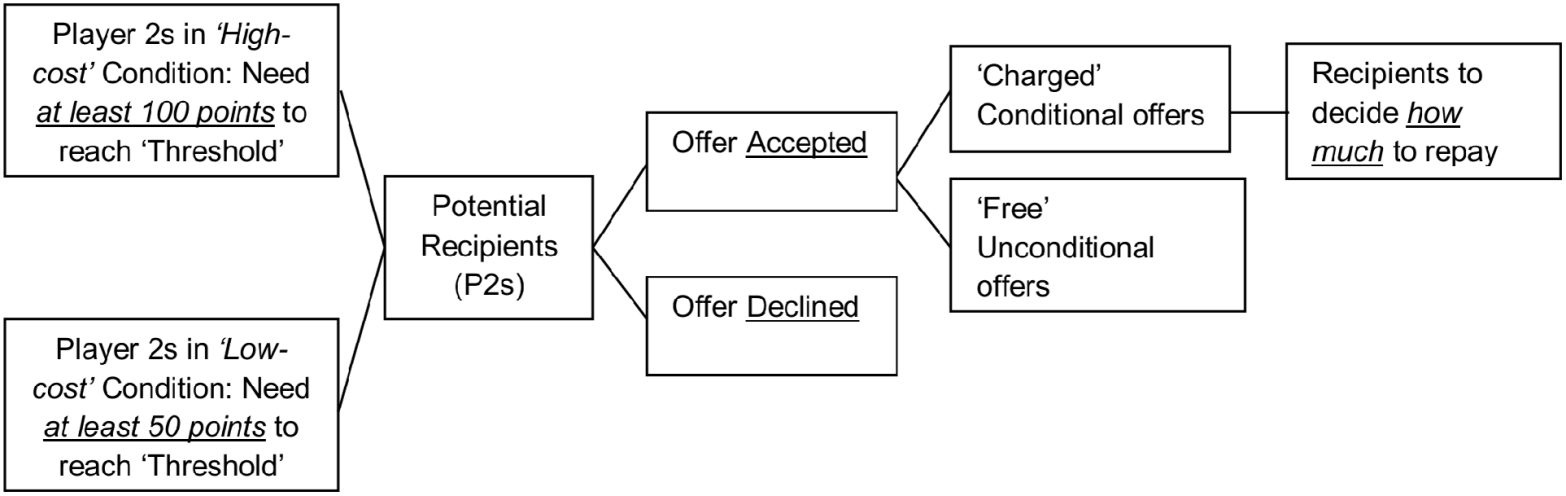
The flow of decisions made by a potential recipient (Player 2).

We checked that the recipients of conditional offers understood the game and that they were always better off economically accepting (rather than declining) the offers, even if they fulfilled the repayment. P2s who accepted their conditional offers were then shown a reminder of their expected repayment. Immediately after that reminder P2s decide whether to repay and how much (Fig. 2). Importantly by giving P2s the freedom to decide whether to honour the agreement we created the option of defaulting in terms of a ‘breach of contract’ [48]. P2s (who received conditional offers) were initially unaware of this latter option to default, when making their emotional judgements so as not to confound their emotional judgements, which are the primary outcome in this study, and make these judgements comparable to the unconditional condition where there is no option to repay. P2s who received unconditional offers were not given a latter option to repay the ‘free’ gift. This was to reflect the real world situation whereby a free gift from a stranger without the option to repay (e.g., altruistic living organ donation, blood donation) would remain as such. However, when there is an explicit expectancy to repay there is always the implicit fear for the donor (P1s) that the recipient will default with a ‘breach of contract’ even if that was not their initial intention. Thus by offering the recipients an opportunity to penalise their ‘donors’ for their repayment request, we provided a behavioural measure of positive (full repayment) and negative reciprocity (zero back-transfer or partial repayment) and the level of default gives a behavioural index of how the recipients (who accepted their conditional offers) valued the violation on the injunctive norm. Thus the option to show defecting behaviour provides a secondary outcome and one that does not bias the initial emotional judgements.

In this game P1s’ final payoffs were affected by their P2 partners’ actual acceptance and repayment decisions. That is, any cooperation or defaulting by P2s would directly impact on their respective P1 partners’ final payoffs. All of the P2s’ decisions (e.g. acceptance, repayment) were fed back to their P1 partners. Afterwards, all participants were shown onscreen their respective compensations. We then asked all participants to complete the Marlowe-Crowne Social Desirability Scale (MCSD, [49]). In so doing we intended to address any potential validity concerns attributable to Socially Desirable Responding (SDR) [50], [51]. That is people trying to present their self-report responses in a favourable manner [52]. The session ended here and the participants were debriefed and paid.

### Ethics Statement

This study was approved by the ethics committee of the School of Psychology at the University of Nottingham, and all participants were older than 17 and all gave written informed consent prior to participation as approved by the ethics committee (Approved 11^th^ Feb, 2013; Ref. Code: 267).

## Results

### Pre-offer Ratings

Both P1s and P2s agreed that their partners’ die-rolling outcomes were chance- instead of ability-governed. See S1a Table and S1b Table in S1 Supporting Information, for further details. These attributional tendencies were not influenced by (1) gender, (2) participants’ assigned role (P1s vs. P2s) and (3) assigned cost conditions. Meanwhile, a paired-samples T-Test indicated a significant difference, (t (121) = 14.82, *p*<.001), between participants’ average ‘Chance’ (M: 5.04) and ‘Ability’ attributions (M: 1.59). This suggested that participants in this game were generally more inclined to interpret their partners’ good (or mis-) fortune to chance rather than to ability.

### Offers Made, Post-offer Ratings and Socially Desirable Responding (SDR) Measures

There were 49 P2s (80%) who received an offer and of these 24 received a conditional (10 received a ‘Full Repayment’, 8 a ‘Partial Repayment’ and 6 an ‘Interest Repayment’ offer) and 25 an unconditional offer. While there was one instance of rejection among the recipients of unconditional offers (rejection rate: 1/25 = 4%: 96% acceptance), three recipients of conditional offers declined theirs (rejection rate: 3/24 = 12.5%: 87.5% acceptance). Neither cost of helping nor gender significantly differentiated (1) the number of P1s who agreed to help (conditionally or unconditionally, N = 49), (2) the conditionality of offers P1s made, (3) helpers’ (N = 49) preferred repayment options and (4) P2s’ (N = 61) the post offer ratings. See S2a Table & S2b Table in S2 Supporting Information, and S3a Table and S3b Table in S3 Supporting Information for details. As such, we collapsed findings from both cost conditions and genders for the present analysis.

Independent samples T-Tests were conducted to examine if the conditionality of offer alone, elicited differential emotions toward donors and interpretations of donors’ intent in recipients. As illustrated in Table 1, recipients of unconditional offers reported (1) more gratitude (p<.05), (2) that the donors were genuinely more helpful (p<.05), and (3) a higher tendency to reciprocate (p<.01) in future exchanges.

**Table 1:** Statistics for P2s’ post-offer ratings.

			Types of offer received			
			Unconditional^a^	Conditional^b^		
	*t-* *statistic*	*df*	*Mean* *(SD)*	*Mean* *(SD)*	*Mean* *Difference*	*Std. Error* *Difference*
State Annoyance	1.729	37.9	1.40(0.71)	1.88(1.15)	0.475	0.275
State Gratitude	2.046*	47	6.30(1.27)	5.63(1.02)	0.675	0.330
State Indebtedness	0.895	47	4.96(1.72)	4.46(2.19)	0.502	0.561
Perceived PartnerGenuine Helpfulness	2.014*	47	5.80(1.19)	5.13(1.15)	0.675	0.335
Obligation to Repay	1.828	47	4.44(1.73)	5.33(1.69)	0.893	0.489
Reasonableness ofP1s’ Decisions	1.817	47	4.72(1.62)	5.46(1.18)	0.738	0.406
Perceived LowCost of Help	1.880	47	3.76(1.79)	4.67(1.58)	0.907	0.482
Reciprocating Tendency	2.736**	38.6	6.28(0.79)	5.46(1.25)	0.822	0.300

*Note*. ^a^N = 25. ^b^N = 24. df = Degree of Freedom.

*p<.05 (two-tailed);

**p<.01 (two-tailed).

**Figure 3:**
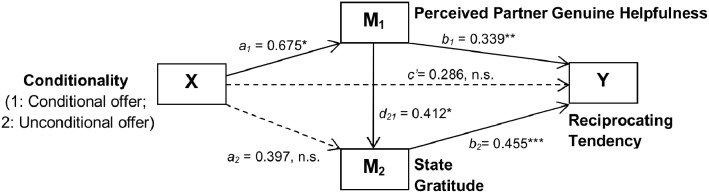
A statistical diagram of the serial multiple mediator model for the Donor Intent Perception. Legends: Note. N = 49; n.s. = Not Significant; *p<.05; **p<.01; ***p<.001.

A serial multiple mediator model was specified [46] (Fig. 3). This model examines if the effect of offer conditionality on reciprocity tendency is mediated serially by (1) the degree that the donor was perceived as genuinely helpful and (2) the recipients level of gratitude. We adopted the Bootstrap Confidence Intervals (CI) approach as we analyse the hypothesized path model. Hayes [53] argued that bootstrapping is ‘particularly useful relative to normal approach (i.e. the Sobel test [54]) in smaller samples (pp.110)’ as it better handles the non-normality (or irregularity) of the sampling distribution (of the indirect effects under scrutiny) which is inherent in smaller samples, thus rendering ‘a test with higher power (pp.106)’. The results are presented in Table 2 and Fig. 3. The results show that the conditionality of an offer indirectly influenced the recipients’ tendency to reciprocate through its effect on attributions of helpfulness and gratitude towards the donor. Recipients receiving an unconditional offer were more prone to perceive their donor (helper) as genuinely helpful (a_1_ = 0.675, *p*<.05), and therefore reported more gratitude (d_21_ = 0.412, *p*<.01). This elevated gratitude predicts a greater eagerness to reciprocate (b_2_ = 0.455, *p*<.001). A bias-corrected bootstrap CI for this total indirect effect (a_1_d_21_b_2_ = 0.675*0.412*0.455 = 0.1266) based on 5,000 samples did not include zero (0.0119 to 0.3682), indicating a statistically significant indirect effect [55]. Additional analyses indicated that receiving an unconditional offer indirectly influenced gratitude through attributions of helpfulness, (the bootstrap CI for this effect (a_1_d_21_) did not include zero (0.0266 to 0.5867). Meanwhile, there was no evidence that (1) receiving an unconditional offer influenced reciprocity tendency directly (c’ = 0.2857, *p* = .188), or that (2) receiving an unconditional offer influenced reciprocity indirectly through gratitude only, as the bootstrap CI for this indirect effect (a_2_b_2_) included zero (–0.1437 to 0.6326).

**Table 2:** Regression Coefficients, Standard Errors, and Model Summary Information for the Donor Intent Perception Serial Multiple Mediator Model.

						Consequent							
		M_1_ (Perceived PartnerGenuine Helpfulness, PGH)				M_2_ (State Gratitude, Gs)					Y (Tendency toReciprocate, Rec)		
Antecedent		Coeff.	*SE*	*p*		Coeff.		*SE*	*p*		Coeff.	*SE*	*p*
X(Cond*)	*a_1_*	.675	.335	.0497	*a_2_*	.397	.316		.215	*c’*	.286	.214	.188
M_1_(PGH)		—	—	—	*d_21_*	.412	.132		.003	*b_1_*	.339	.097	.0010
M_2_(Gs)		—	—	—		—	—		—	*b_2_*	.455	.098	<.0001
Constant	*iM_1_*	4.45	.533	<.001	*iM_2_*	3.12	.759		<.001	*i_Y_*	.876	.591	.145
		*R^2^* = .080				*R^2^* = .243					*R^2^* = .622		
		*F* (1,47) = 4.058, *p* = .0497				*F* (2,46) = 7.368, *p* = .0017					*F* (3,45) = 24.71, *p*<.001		

Note. *Conditionality: ‘1’denotes receipt of a conditional offer while ‘2’ denotes receipt of an unconditional offer.

Associations of Marlowe-Crowne Social Desirability Scale (MCSD) scores with the main study variables revealed no significant correlations with most of the recipients’ ratings and donors’ helping decisions. See S4a Table and S4b Table in S4 Supporting Information, for details. This suggested that social desirability response set did not have substantially contaminated the current analysis.

### Behavioural Data

Twenty-one recipients who had accepted their conditional offers were asked to repay. Of all these 21 recipients eight (i.e. 38.1%) defaulted by either repaying partially or nothing, while the rest (N = 13, 61.9 %) all repaid fully as agreed. Interestingly, scrutiny of these eight defaulters response revealed one instance of zero repayment, while the rest (N = 7) on average repaid 58.4% of their expected repayment. A Wilcoxon Signed-ranked test demonstrated a significant discrepancy, (Z = 2.53, *p* = .012), between the defaulter’s ‘Expected Repayment’ (M: 86.6) and his/her ‘Actual Repayment’ (M: 38.9). This suggested that a significant level of defaulting occurred among these conditional recipients and that is attributable to those 8 defaulters. There was, however, no evidence that the level of conditionality influenced the number of P2s who (1) accepted (or declined) their conditional offers, and (2) repaid fully or defaulted (See S5 Table in S5 Supporting Information for details). This indicated that whether the recipients of conditional offers were treated relatively more ‘harshly’ over one another neither elicited (1) more acceptances or rejections of offers, nor (2) more cooperation or defecting.

## Discussion

The present data illustrated that conditionality of an offer of help alone, when the recipient is not aware of the options open to the helper, can significantly influence how recipients interpreted the helpers’ intentions, as well as the recipients’ feelings of gratitude, and their tendency to be willing to reciprocate. The results show that the greater the perceptions of the donors’ intentions to be helpful (influenced by conditionality of the offer) the greater the level of gratitude towards them, and resulting in a greater tendency to reciprocate. Furthermore, the behavioural data revealed that when recipients received a conditional offer, nearly 40% of them (8 out of 21) acted uncooperatively despite acceptance, either giving less than they agreed or defecting completely. This indicated that people, albeit lacking awareness of the options open to donors, still made differential judgments based solely on whether they received a conditional or unconditional offer. We interpret this as an example of the use of injunctive normative beliefs about fairness. That is, in the absence of all information about the donor, recipients could only infer their donors’ intent to be helpful with reference to the normative beliefs about what an average ‘fair’ individual *should do* when their opportunity to be in a position to help was determined by chance.

Furthermore, analyses show that people’s tendency to want to reciprocate when treated fairly and be less cooperative when treated less fairly in a normative sense, is, in part, dependent on attributional and emotional processing. People who inferred that the donor’s intentions were good, –feel gratitude towards the donor - were more likely to be eager to help them back in the future. Thus intentions and gratitude are two key processes that help account for the link between conditionality of offers and willingness to reciprocate. Importantly, these results show that injunctive normative beliefs about fairness are important with respect to intentions [29], [32] and emotional responses associated with cooperation and reciprocation [14], [16]. That is, our recipients had no knowledge about who their helpers were, what their helpers had done in the past, would never meet them and crucially knew nothing about the choices of offer that were open to the helpers. All they knew was that their helpers’ good fortune to be in a position to help was by pure chance. Consistent with the idea that under these circumstances people should act generously and help for free, we show clearly that when this is not the case and conditions are imposed by some helpers the recipients view these helpers as less genuinely helpful, feel less gratitude and are less likely to want to reciprocate to them. The lack of willingness to reciprocate resonates with the indirect reciprocation literature which shows that we prefer to help ‘kind’ people with a good reputation [56], [57]. Here we show that reputation can be inferred from very minimal information – the conditionality of help only.

While only a relatively small fraction of recipients received conditional offers a non-negligible percentage (38%) of them still showed uncooperative behaviour, and interestingly all but one of these defectors chose to be somewhat ‘altruistic’ by repaying the majority of their obligated repayment. It is, important to investigate the psychological or situational factors that motivate someone to engage in this kind of ‘impure’ free-riding or ‘cheap-riding’ [58]–[60]. Such cheap-riding may reflect dissatisfaction with the donors, the actual value the recipients place on the offer or perhaps a means to irk the donors by deliberately underpaying on their agreed repayment. This reduced payback has been documented before in trust games when the recipient knew a priori that the helper could have decided not to make their investment conditional (or punishable) [7]. Here we observe the same behaviour in the absence of this knowledge. This has been interpreted as evidence for ‘strong reciprocity’ [7], [61]. But why pay back at a reduced level at all? The recipients in our study, if they wanted to, could have kept all the money (the situation was anonymous with no repercussions); however, it appears that they chose to signal their disapproval directly to the helpers by actively under-repaying. We interpret this as a means of giving the helpers their ‘just deserts’ and signally disapproval designed to make the helper act more generously in the future [62]. That is, taking everything could annoy the helper and the helper may be able to dismiss that recipient’s choice not to repay as the recipient being an ‘exception’ and not representative of others, in which case they may still offer help in the future. It may also result in anger toward the recipient that generalizes to others hence reduced their willingness to help in the future. Thus not repaying may have a number of consequences. However, partial repayment signals to the helper that they were not generous in the first place – did not observe the injunctive norm – by indicating via partial repayment what they believed the (conditional) help was really worth. This therefore, may serve to make the helper feel guilty. These feeling of guilt are likely to make the helpers perhaps more cooperative in the future [63]–[65]. Thus we feel that partial repayment is a means to reinforce fairness norms. Nevertheless, at present we could not rule out any alternative perspectives (e.g. the recipients were being nasty) that could account for partial repayment.

There are a number of interesting observations about the unconditional offers. First, in the context of ‘free’ gift we observe that most recipients accepted (96% acceptance rate 24/25 for ‘free’ compared to 87.5% 21/24 for the ‘charged’ gifts) rather that declined the ‘free’ gift because of suspicion [66] or disapproval of these overly generous helpers [67], [68]. This may be because in the context of the unconditional offer the helper was (1) anonymous, (2) there was by definition no obligation to repay and (3) the helper’s good fortune to be in a position to help was by chance.

## Supporting Information

S1 Supporting Information **Statistical Analyses of Participants’ Attributions of Partners’ Desirable (or Poor) Die-rolling Outcomes.**
(DOCX)

S2 Supporting Information **Effecs of Cost of Helping.**
(DOCX)

S3 Supporting Information **Effects of Gender.**
(DOCX)

S4. Supporting Information **Effects of Social Desirability Responding.**
(DOCX)

S5 Supporting Information **Effect of Level of Conditionality on Recipients’ (of Conditional Offers) Decision Making.**
(DOCX)

S6 Supporting Information **More Statistics for Player 2s’ Post-offer Ratings.**
(DOCX)

S7 Supporting Information **On-screen instructions for the game.**
(DOCX)

S8 Supporting Information **Calculation of Participants’ Compensations.**
(DOCX)
