# Supplementary material for: Gratefully Received, Gratefully Repaid: The Role of Perceived Fairness in Cooperative Interactions
Source: PLoS One. 2014 Dec 8;9(12):e114976. doi: 10.1371/journal.pone.0114976 (PMC4259482; doi:10.1371/journal.pone.0114976)
Supplement: S1 Supporting Information — Statistical Analyses of Participants’ Attributions of Partners’ Desirable (or Poor) Die-rolling Outcomes. (DOCX) [file pone.0114976.s001.docx]

**Supporting Information 1: Statistical Analyses of Participants’ Attributions of Partners’ Desirable (or Poor) Die-rolling Outcomes**

We intended to check, via two pre-offer ratings using 7-point Likert Type Scales (1= ‘Not At All’; 7 = ‘Completely’), if the present die-rolling procedure succeeded in leading our participants (both Player 1s and Player 2s) to believe that what segregated the ‘winners’ (i.e. making the 200 point ‘threshold’) from the ‘losers’ (i.e. missing that) in the game was chance rather than ability.

As illustrated in Table S1a, participants in general (M: 5.04, SD: 2.21) were convinced that their partners’ high or low die-rolling scores were due to good (or bad) luck. The independent samples t-tests indicated that this high propensity to impute partner’s outcomes to chance was irrelevant to (1) gender, (2) which cost condition and (3) which role the participant was assigned to. On the contrary, Table S1b shows that overall participants were reluctant to attribute their partners’ high or low die-rolling scores to their abilities (M: 1.59, SD: 1.22). Likewise, this attributional tendency, according to the results of the independent samples t-tests, has nothing to do with (1) gender, (2) which cost condition and (3) which role the participant was assigned to.

Table S1a. Statistics for Participants’ Pre-offer rating: Item ‘Chance’ Attribution

|  | ‘Chance’ Attribution ^1^ | | | | | | | | |  |
| --- | --- | --- | --- | --- | --- | --- | --- | --- | --- | --- |
|  | | | *N* | *Mean* | *SD* | *SE* | *df* | *Mean Difference* | *t-statistic* | *p (two-tailed)* |
| Gender | | Male | 50 | 4.76 | 2.37 | 0.34 |  |  |  |  |
|  | | Female | 72 | 5.24 | 2.08 | 0.25 |  |  |  |  |
|  | | Total | 122 | 5.04 | 2.21 | 0.20 | 96.4 | 0.476 | 1.147 | 0.254 |
| Condition | | Low-cost | 62 | 5.26 | 2.20 | 0.28 |  |  |  |  |
|  | | High-cost | 60 | 4.82 | 2.21 | 0.29 |  |  |  |  |
|  | | Total | 122 | 5.04 | 2.21 | 0.20 | 120 | 0.441 | 1.106 | 0.271 |
| Role | | Player 1s | 61 | 4.80 | 2.29 | 0.29 |  |  |  |  |
|  | | Player 2s | 61 | 5.28 | 2.11 | 0.27 |  |  |  |  |
|  | | Total | 122 | 5.04 | 2.21 | 0.20 | 120 | 0.475 | 1.192 | 0.235 |

*Note.* ^1^ ‘To what extent you think that your partner’s high (low) score is attributable to his/her good (bad) luck’. The item was measured on 7-point Likert Type Scales (1= ‘Not At All’; 7 = ‘Completely’).

Table S1b. Statistics for Participants’ Pre-offer rating: Item ‘Ability’ Attribution

|  | ‘Ability’ Attribution ^1^ | | | | | | | | |  |
| --- | --- | --- | --- | --- | --- | --- | --- | --- | --- | --- |
|  | | | *N* | *Mean* | *SD* | *SE* | *df* | *Mean Difference* | *t-statistic* | *p (two-tailed)* |
| Gender | | Male | 50 | 1.58 | 1.11 | 0.16 |  |  |  |  |
|  | | Female | 72 | 1.47 | 1.01 | 0.12 |  |  |  |  |
|  | | Total | 122 | 1.59 | 1.22 | 0.11 | 120 | 0.108 | 0.558 | 0.578 |
| Condition | | Low-cost | 62 | 1.55 | 1.28 | 0.16 |  |  |  |  |
|  | | High-cost | 60 | 1.63 | 1.16 | 0.15 |  |  |  |  |
|  | | Total | 122 | 1.59 | 1.22 | 0.11 | 120 | 0.085 | 0.384 | 0.702 |
| Role | | Player 1s | 61 | 1.38 | 0.86 | 0.11 |  |  |  |  |
|  | | Player 2s | 61 | 1.80 | 1.47 | 0.19 |  |  |  |  |
|  | | Total | 122 | 1.59 | 1.22 | 0.11 | 96.7 | 0.426 | 1.955 | 0.053 |

*Note.* ‘To what extent you think that your partner’s high (low) score is because of his/her capability (incompetence)’. The item was measured on 7-point Likert Type Scales (1= ‘Not At All’; 7 = ‘Completely’).

A paired-samples t-test indicated a highly significant difference, (t (121) = 14.82, *p* <.001), between participants’ average ‘Chance’ (M: 5.04) and ‘Ability’ attributions (M: 1.59). This suggested that participants in general were evidently more prone to attribute their partners’ good (or mis-) fortune to chance than to ability. We are, nevertheless, interested to find out if this ‘attributional bias’ was explainable participant’s gender, assigned role or cost condition. Thus the dependent variable would be the *net difference* between each participant’s ‘Chance’ and ‘Ability’ attribution ratings. Again, as illustrated in Table S1c, independent samples T-Tests revealed that this attributional bias were neither explainable by (1) gender, (2) assigned role, nor (3) cost condition he/she was in. This implied that the participants overall appeared to have a consensus over how to interpret partners’ die-rolling outcomes.

Table S1c. Statistics for Participants’ Attributional Bias

|  | ‘Attributional Bias’ ^1^ | | | | | | | | |  |
| --- | --- | --- | --- | --- | --- | --- | --- | --- | --- | --- |
|  | | | *N* | *Mean* | *SD* | *SE* | *df* | *Mean Difference* | *t-statistic* | *p (two-tailed)* |
| Gender | | Male | 50 | 3.06 | 2.90 | 0.41 |  |  |  |  |
|  | | Female | 72 | 3.72 | 2.30 | 0.27 |  |  |  |  |
|  | | Total | 122 | 3.45 | 2.57 | 0.23 | 89.6 | 0.662 | 1.348 | 0.181 |
| Condition | | Low-cost | 62 | 3.71 | 2.55 | 0.32 |  |  |  |  |
|  | | High-cost | 60 | 3.18 | 2.59 | 0.33 |  |  |  |  |
|  | | Total | 122 | 3.45 | 2.57 | 0.23 | 120 | 0.526 | 1.132 | 0.260 |
| Role | | Player 1s | 61 | 3.43 | 2.51 | 0.32 |  |  |  |  |
|  | | Player 2s | 61 | 3.48 | 2.66 | 0.34 |  |  |  |  |
|  | | Total | 122 | 3.45 | 2.57 | 0.23 | 120 | 0.049 | 0.105 | 0.916 |

*Note.* ^1^ Attributional Bias = Participant’s ‘Chance’ Attribution – ‘Ability’ Attribution Rating
