# Supplementary material for: Gratefully Received, Gratefully Repaid: The Role of Perceived Fairness in Cooperative Interactions
Source: PLoS One. 2014 Dec 8;9(12):e114976. doi: 10.1371/journal.pone.0114976 (PMC4259482; doi:10.1371/journal.pone.0114976)
Supplement: S2 Supporting Information — Effecs of Cost of Helping. (DOCX) [file pone.0114976.s002.docx]

**Supporting Information 2: Effects of Cost of Helping**

We examined the effects cost (i.e. Low-cost vs. High-cost Helping) had significantly influenced 1) P2s’ post-offer ratings, 2) repayment decisions for those who received a conditional offer, 3) donors’ excess transfers and their 4) other helping-related decisions

Table S2a. Effect of Cost of Helping on Player 2s’ 1) post-offer ratings, 2) repayment decisions; and on 3) Donors’ Transfer Decisions

|  |  |  | Testing Condition | |  | | |  |
| --- | --- | --- | --- | --- | --- | --- | --- | --- |
|  |  | Low-cost | | High-cost | |  | | |
|  | *t-statistic* | *df* | *Mean (SD)* | *Mean (SD)* | *Mean Difference* | | *p-value (two-tailed)* | |
| 1. P2s’^1^ Post-offer Ratings |  |  |  |  |  | |  | |
| State Annoyance | 0.540 | 59 | 2.29 (1.77) | 2.07 (1.43) | 0.224 | | .591 | |
| State Gratitude | 1.059 | 59 | 4.79 (2.28) | 5.37 (1.96) | 0.576 | | .294 | |
| State Indebtedness | 1.059 | 59 | 3.68 (2.20) | 4.30 (2.40) | 0.623 | | .294 | |
| Perceived Genuine Helpfulness^2^ | 0.406 | 47 | 5.54 (1.10) | 5.40 (1.32) | 0.142 | | .686 | |
| Obligation to Repay^2^ | 1.156 | 47 | 4.58 (1.69) | 5.16 (1.80) | 0.577 | | .253 | |
| Reasonableness of Donors’ Decisions | 0.487 | 52.6 | 5.10 (1.30) | 4.90 (1.81) | 0.197 | | .628 | |
| Perceived Low Cost of Help^2^ | 1.348 | 47 | 4.54 (1.62) | 3.88 (1.81) | 0.662 | | .184 | |
| Reciprocating Tendency | 0.226 | 59 | 5.19 (1.85) | 5.30 (1.82) | 0.106 | | .822 | |
| 2. Recipients’ ^3^ Repayment (in points) |  |  |  |  |  | |  | |
| Actual Repayment | 1.853 | 13.8 | 49.0 (17.3) | 73.7 (40.4) | 24.7 | | .085 | |
| Discrepancy between Actual and Expected Repayment | 0.973 | 12.5 | 10.0 (17.0) | 25.6 (50.2) | 15.6 | | .349 | |
| 3. Donors’ ^4^ Transfer (in points) |  |  |  |  |  | |  | |
| Discrepancy between Actual and Expected (i.e. Minimum) Transfer | 1.383 | 41.3 | 14.4 (21.9) | 6.84 (15.5) | 7.54 | | .174 | |

*Note.* ^1^ There were 31 Player 2s in ‘High-cost’ condition of which 25 received either a conditional or unconditional offer while 24 out of 30 Player 2s in ‘Low-cost’ condition received either a conditional or unconditional offer.
^2^ Only P2s who received either a conditional or unconditional offer (N=49) were required to respond to this item.
^3^ Twenty-one recipients of conditional offers were expected to repay, 10 of them were from ‘Low-cost’ condition and 11 were from the ‘High-cost’ condition.
^4^There were 24 Player 1s from the ‘Low-cost’ condition and 25 from the ’High-cost’ condition who decided to help either conditionally or unconditionally.

Independent-sample T-Tests revealed that cost condition failed to significantly differentiate any of 1) the recipients’ post-offer ratings, repayment decisions of the recipients of conditional offers and 3) the donors’ magnitude of ‘over-donation’. The results are presented in Table S2a.

Table S2b. Effect of Cost of Helping on Donors’ Helping Decisions.

|  |  | Testing Condition | |  |  |  |
| --- | --- | --- | --- | --- | --- | --- |
|  | Low-cost | | High-cost |  |  |  |
|  |  | *N* | *N* | *Chi-square* | *df* | *p-value (two-tailed)* |
| Donors’ Helping Decisions |  |  |  |  |  |  |
| 1.To Help or Not to Help | Helped | 24 | 25 |  |  |  |
|  | Not Helped | 7 | 5 |  |  |  |
|  | Total | 31 | 30 | .337 | 1 | .561 |
| 2. Conditionality of offer Made | Unconditional | 13 | 12 |  |  |  |
|  | Conditional | 11 | 13 |  |  |  |
|  | Total | 24 | 25 | .186 | 1 | .666 |
| 3. Preferred Repayment Modes | Partial | 5 | 5 |  |  |  |
|  | Full | 3 | 5 |  |  |  |
|  | Interest | 3 | 3 |  |  |  |
|  | Total | 11 | 13 | N.A.^1^ | | |

*Note* ^1^ Pearson’s Chi-squared test was not conducted for ‘Preferred Repayment Modes’ as more than 20% of the cells have expected count less than 5. We instead ran the Fisher’s Exact Test, and the results revealed that donors’ preferred repayment modes did not significantly differ by Testing (i.e. Cost of Helping) Condition (p= .882, two-tailed) .

Additionally, a Chi-square test was used to determine whether there was a significant difference between donors from ‘Low-cost’ and ‘High-cost’ condition in their 1) decisions to help (or not), and 2) conditionality (need to repay or not) of offers made should they agreed to help. As illustrated in Table S2b, a contingency Chi-square test revealed that neither decisions significantly differed by testing condition. Furthermore, the Fisher’s exact test was conducted to determine if donors’ preferences of repayment modes would differ by testing condition. The results indicated no significant effect of testing condition on (conditional) donors’ choices of repayment modes.
