# Supplementary material for: Gratefully Received, Gratefully Repaid: The Role of Perceived Fairness in Cooperative Interactions
Source: PLoS One. 2014 Dec 8;9(12):e114976. doi: 10.1371/journal.pone.0114976 (PMC4259482; doi:10.1371/journal.pone.0114976)
Supplement: S3 Supporting Information — Effects of Gender. (DOCX) [file pone.0114976.s003.docx]

**Supporting Information 3: Effects of Gender**

We examined if participants’ gender had significantly influenced 1) recipients’ post-offer ratings, 2) repayment decisions for those who received a conditional offer, 3) donors’ transfer decisions, and their 4) other helping-related decisions.

Table S3a. Effect of Gender on Recipients’ 1) Post-offer ratings, 2) Repayment decisions; and on 3) Donors’ Transfer Decisions

|  |  | Participants’ Gender | | |  |  |
| --- | --- | --- | --- | --- | --- | --- |
|  |  | Male | | Female | |  |
|  | *t-statistic* | *df* | *Mean (SD)* | *Mean (SD)* | *Mean Difference* | *p-value (two-tailed)* |
| 1. P2s’^1^ Post-offer Ratings |  |  |  |  |  |  |
| State Annoyance | 0.439 | 59 | 2.05 (1.40) | 2.24 (1.71) | 0.194 | .662 |
| State Gratitude | 0.513 | 59 | 5.28 (2.14) | 4.98 (2.14) | 0.299 | .610 |
| State Indebtedness | 0.789 | 59 | 3.65 (2.23) | 4.15 (2.34) | 0.496 | .433 |
| Perceived Genuine Helpfulness^2^ | 0.122 | 47 | 5.50 (1.32) | 5.45 (1.18) | 0.045 | .903 |
| Obligation to Repay^2^ | 0.179 | 47 | 4.81 (1.97) | 4.91 (1.67) | 0.097 | .859 |
| Reasonableness of Donors’ Decisions | 2.161 | 59 | 5.60 (1.39) | 4.71 (1.57) | 0.893 | .035* |
| Perceived Low Cost of Help^2^ | 0.925 | 47 | 3.88 (1.75) | 4.36 (1.73) | 0.489 | .360 |
| Reciprocating Tendency | 0.758 | 59 | 5.50 (1.67) | 5.12 (1.90) | 0.378 | .452 |
| 2. Recipients’ ^3^ Repayment (in points) |  |  |  |  |  |  |
| Actual Repayment | 0.401 | 19 | 66.7 (42.7) | 60.1 (30.3) | 6.60 | .693 |
| Discrepancy between Actual and Expected Repayment | 1.160 | 19 | 33.3 (47.2) | 12.1 (33.9) | 21.2 | .260 |
| 3. Donors’ ^4^ Transfer (in points) |  |  |  |  |  |  |
| Actual Transfer | 0.924 | 47 | 90.0 (29.9) | 82.5 (27.2) | 7.54 | .360 |
| Discrepancy between Actual and Expected (i.e. Minimum) Transfer | 0.315 | 47 | 9.61 (18.3) | 11.4 (20.2) | 1.74 | .755 |

*Note.* ^1^ There were 16 out of 20 male P2s and 33 out of 41 female P2s who received either a conditional or unconditional offer.
^2^ Only P2s who received either a conditional or unconditional offer (N=49) were required to respond to this item. ^3^ Twenty-one recipients of conditional offers were expected to repay, there were 6 male recipients and 15 female recipients.
^4^ There were 23 male donors and 26 female donors. *p < .05 (two-tailed).

Independent-sample T-Tests revealed that gender failed to significantly differentiate any of 1) the recipients’ post-offer ratings (except that male P2s rated their partners’ decisions as more ‘reasonable’ compared to their female counterparts), 2) repayment decisions of the recipients of conditional offers and 3) the donors’ magnitude of transfers and ‘over-donation’, i.e. the excess amount of transfer relation to the minimum requirement,. The results are presented in Table S3a.

Table S3b. Effect of Gender on Donors’ Helping Decisions.

|  | Participants’ Gender | | |  |  |  |
| --- | --- | --- | --- | --- | --- | --- |
|  |  | Male | Female |  |  |  |
|  |  | *N* | *N* | *Chi-square* | *df* | *p-value (two-tailed)* |
| Donors’ Helping Decisions |  |  |  |  |  |  |
| 1.To Help or Not to Help | Helped | 23 | 26 |  |  |  |
|  | Not Helped | 7 | 5 |  |  |  |
|  | Total | 30 | 31 | .501 | 1 | .534 |
| 2. Conditionality of offer made | Unconditional | 10 | 15 |  |  |  |
|  | Conditional | 13 | 11 |  |  |  |
|  | Total | 23 | 26 | .987 | 1 | .396 |
| 3. Preferred Repayment Modes | Partial | 5 | 5 |  |  |  |
|  | Full | 4 | 4 |  |  |  |
|  | Interest | 4 | 2 |  |  |  |
|  | Total | 13 | 11 | N.A.^1^ | | |

*Note* ^1^ Pearson’s Chi-squared test was not conducted for ‘Preferred Repayment Modes’ because more than 20% of the cells have expected count less than 5. We instead ran the Fisher’s Exact Test, and the results revealed that donors’ preferred repayment modes did not significantly differ by Gender (p= .769, two-tailed)

Additionally, a contingency Chi-square test was used to determine whether there was a significant difference between female and male donors in their 1) decisions to help (or not), and 2) conditionality of offers made should they agreed to help. As illustrated in Table S3b, the Chi-square test revealed that neither decisions significantly (p >.35) differed by gender. Furthermore, the Fisher’s exact test was conducted to determine if donors’ preferences of repayment modes would differ by gender. The results indicated no gender differences (p >.75) in conditional donors’ selections of repayment modes.
