# Supplementary material for: Gratefully Received, Gratefully Repaid: The Role of Perceived Fairness in Cooperative Interactions
Source: PLoS One. 2014 Dec 8;9(12):e114976. doi: 10.1371/journal.pone.0114976 (PMC4259482; doi:10.1371/journal.pone.0114976)
Supplement: S4. Supporting Information — Effects of Social Desirability Responding. (DOCX) [file pone.0114976.s004.docx]

**Supporting Information 4: Effects of Social Desirability Responding**

We intended to check whether Socially Desirable Responding (SDR) had systematically affected recipients’ post-offer ratings (Table S4a), repayment decisions for those who received a ‘charged’ conditional offer (Table S4a), and donors’ range of helping-related decisions (Table S4b). We operationalised participants’ SDR tendencies as their composite scores of the Marlowe-Crowne Social Desirability Scale (MCSD)

Table S4a. Effect of Socially Desirable Responding (Measured using Marlowe-Crowne Social Desirability Scale, Crowne & Marlowe, 1962) on recipients’ post-offer ratings and repayment decisions

|  | | Gs | As | Is | Os | Reas | PGH | Cost | Rec | AR | UR |
| --- | --- | --- | --- | --- | --- | --- | --- | --- | --- | --- | --- |
| MCSD | Pearson Correlation (r) | .089 | -.142 | -.308 | -.007 | .141 | -.073 | .098 | .159 | .224 | -.087 |
|  | P-value (2-tailed) | .541 | .329 | .031* | .964 | .332 | .620 | .502 | .274 | .329 | .706 |
|  | N | 49 | 49 | 49 | 49 | 49 | 49 | 49 | 49 | 21 | 21 |

*Note.* MCSD: Marlowe-Crowne Social Desirability Scale; Gs = State Gratitude; As = State Annoyance; Is= State Indebtedness; Os = Perceived Obligation to Repay; Reas = Perceived Reasonableness of Partners’ decision; PGH = Perceived Genuine Helpfulness in partners’ help; Cost= Perceived Low Cost of Partners’ Help; Rec = Recipients’ Eagerness to Reciprocate; AR = Actual Repayment by Recipients of Conditional offers; UR = Subtractions of ‘Expected Repayment’ from ‘Actual Repayment’ by recipients who accepted their conditional offers (negative values denote defaulting). * p <.05 (two-tailed)

As illustrated in Table S4a, there was no evidence that 1) SDR tendency was associated with most of the recipients’ (N=49) post-offer ratings or that 2) SDR tendency influenced the repayment decisions

Table S4b. Effect of Socially Desirable Responding on donors’ helping decisions

|  | | Help | Conditionality | Tran^1^ | OD | RM |
| --- | --- | --- | --- | --- | --- | --- |
| MCSD | Pearson Correlation (r) | -.095 | -.121 | .290* | .147 | .399 |
|  | P-value (2-tailed) | .468 | .409 | .043 | .313 | .054 |
|  | N | 61 | 49 | 49 | 49 | 24 |

*Note.* MCSD: Marlowe-Crowne Social Desirability Scale; Help = Decision to Help or Not; Conditionality= Unconditional or Conditional offers Made; Trans = Magnitude of Transfer by Donors; OD = Differences between the ‘Actual Transfer’ and ‘Expected (Minimum Obligated) Transfer’ by the Donors; RM = Conditional Donors’ Chosen Repayment Modes (i.e. level of conditionality in offers made)
^1^ This significant correlation disappeared once we controlled for donors’ ‘Expected Transfer’, *r* (46) = .199, p = .175 (two-tailed).
* p <.05 (two-tailed); ** p < .01 (two-tailed).

Meanwhile, SDR tendency was not correlated with the donors’ 1) decision to help or not, 2) conditionality of offers made should they helped, 3) preferences for repayment modes, and 4) magnitude of ‘over-donation’ , i.e. excess amount of transfer in relation to the minimum requirement. The results are presented in Table S4b.

While SDR tendency appeared to be positively (r = .290) and significantly (p <.05) associated with the donors’ magnitude of transfers, this relationship no longer stood (p >.1) once donors’ ‘Expected Transfer’ (i.e. minimum required transfers for the two helping cost conditions) was controlled. This implied that a high (low) transfer is better accounted for by a higher (lower) minimum stipulated transfer rather than a greater (smaller) tendency of an individual donor to be socially desirable.
