# Supplementary material for: Gratefully Received, Gratefully Repaid: The Role of Perceived Fairness in Cooperative Interactions
Source: PLoS One. 2014 Dec 8;9(12):e114976. doi: 10.1371/journal.pone.0114976 (PMC4259482; doi:10.1371/journal.pone.0114976)
Supplement: S5 Supporting Information — Effect of Level of Conditionality on Recipients’ (of Conditional Offers) Decision Making. (DOCX) [file pone.0114976.s005.docx]

**Supporting Information 5: Effect of Level of Conditionality on Recipients’ (of Conditional Offers) decision making**

Table S5 Effect of Level of Conditionality on Player 2s’ (who received conditional offers (N=24)) decision making (acceptance and repayment decisions)

|  | Level of Conditionality | | | |  |  |  |
| --- | --- | --- | --- | --- | --- | --- | --- |
|  |  | Full Repayment | Partial Repayment | Repayment plus 20% Interest |  |  |  |
|  |  | *N* | *N* | *N* | *Fisher’s Exact Test p (2-sided)* | | |
| 1.Offer Acceptance | Accept | 8 | 8 | 5 |  | | |
| (N=24) | Decline | 2 | 0 | 1 |  | | |
|  | Overall | 10 | 8 | 6 | .447 | | |
| 2.Repayment | Cooperate | 6 | 5 | 2 |  | | |
| (N=21) | Default^1^ | 2 | 3 | 3 |  | | |
|  | Overall | 8 | 8 | 5 | .461 | | |

Note. ^1^ We classify ‘defaulters’ as recipients (N=21) who accepted their offers but under-repaid (either repaid partially or nothing)

The Fisher’s exact test was conducted to examine whether the level of conditionality of the conditional offers one received would influence (1) his/her decision to accept or decline the offer, and (2) his/her decision to cooperate (i.e. to fulfil his/her obligated repayment) or to default (i.e. to either repay partially or nothing back). As illustrated in Table S6b, neither decisions were significantly influenced by the level of conditionality of the offers ones received. This highlighted that whether or not the recipients of conditional offers were treated ‘more harshly’, comparatively speaking, over one another did not affect their decisions to accept (or reject) and their decisions to cooperate (or default).
