# Supplementary material for: Gratefully Received, Gratefully Repaid: The Role of Perceived Fairness in Cooperative Interactions
Source: PLoS One. 2014 Dec 8;9(12):e114976. doi: 10.1371/journal.pone.0114976 (PMC4259482; doi:10.1371/journal.pone.0114976)
Supplement: S6 Supporting Information — More Statistics for Player 2s’ Post-offer Ratings. (DOCX) [file pone.0114976.s006.docx]

**Supporting Information 6: More Statistics for Player 2s’ Post-offer Ratings**

Table S6a. Statistics for P2s’ post-offer ratings (Helped P2s versus P2s who were not helped)

|  |  |  | Received an offer? | | | |
| --- | --- | --- | --- | --- | --- | --- |
|  |  |  | Yes ^a^ | No ^b^ |  | |
|  | *t-statistic* | *df* | *Mean (SD)* | *Mean (SD)* | *Mean Difference* | *Std. Error Difference* |
| State Annoyance | 5.23*** | 12.65 | 1.63 (0.97) | 4.42 (1.78) | 2.784 | 0.533 |
| State Gratitude | 19.43*** | 38.41 | 5.97 (1.19) | 1.42 (0.56) | 4.552 | 0.234 |
| State Indebtedness | 13.28*** | 48.00 | 4.71 (1.96) | 1.00 (0.00) ^c^ | 3.714 | 0.280 |
| Reasonableness of P1s’ Decisions | 0.824 | 59 | 5.08 (1.46) | 4.67 (1.97) | 0.415 | 0.504 |
| Reciprocating Tendency | 5.56*** | 12.85 | 5.88 (1.11) | 2.67 (1.92) | 3.211 | 0.577 |

*Note*. ^a^N = 49. ^b^N = 12. ^c^All P2s (N=12) who were not helped rated ‘1 (Not At All)’ for the item ‘I feel indebted to my partner’. SD = Standard Deviation. df = Degree of Freedom. *** p <.001 (two-tailed).

Table S6b. More Statistics for P2s’ post-offer ratings, i.e. P2s who received no offer (N=12) versus Conditionally Helped P2s (N=24) versus Unconditionally Helped P2s (N=25)

|  |  |  | Ratings (1: ‘Not At All’; 7: ‘Completely’) | | | | |  |
| --- | --- | --- | --- | --- | --- | --- | --- | --- |
|  |  | *N* | *Mean* | *SD* | *df* | *F* | *p (two-tailed)* | *ηp^2^* |
| State Annoyance | Not Helped | 12 | 4.42 | 1.78 |  |  |  |  |
|  | Conditionally Helped | 24 | 1.88 | 1.15 |  |  |  |  |
|  | Unconditionally Helped | 25 | 1.40 | 0.71 |  |  |  |  |
|  | Overall | 61 | 2.18 | 1.61 | 2 | 28.98 | <.001^1^ | .500 |
| State Gratitude | Not Helped | 12 | 1.42 | 0.56 |  |  |  |  |
|  | Conditionally Helped | 24 | 5.63 | 1.02 |  |  |  |  |
|  | Unconditionally Helped | 25 | 6.30 | 1.27 |  |  |  |  |
|  | Overall | 61 | 5.07 | 2.13 | 2 | 90.18 | <.001^2^ | .757 |
| State Indebtedness | Not Helped^3^ | 12 | 1.00 | 0.00 |  |  |  |  |
|  | Conditionally Helped | 24 | 4.46 | 2.19 |  |  |  |  |
|  | Unconditionally Helped | 25 | 4.96 | 1.72 |  |  |  |  |
|  | Overall | 61 | 3.98 | 2.30 | 2 | 21.81 | <.001^4^ | .429 |
| Reasonableness of P1s’ Decisions | Not Helped | 12 | 4.67 | 1.97 |  |  |  |  |
|  | Conditionally Helped | 24 | 5.46 | 1.18 |  |  |  |  |
|  | Unconditionally Helped | 25 | 4.72 | 1.62 |  |  |  |  |
|  | Overall | 61 | 5.00 | 1.56 | 2 | 1.76 | .182 | .057 |
| Reciprocating Tendency | Not Helped | 12 | 2.67 | 1.92 |  |  |  |  |
|  | Conditionally Helped | 24 | 5.46 | 1.25 |  |  |  |  |
|  | Unconditionally Helped | 25 | 6.28 | 0.79 |  |  |  |  |
|  | Overall | 61 | 5.00 | 1.56 | 2 | 34.06 | <.001^5^ | .540 |

*Note*. SD = Standard Deviation; df = Degree of Freedom; η_p_^2^ = partial eta squared; MD = Mean Differences.
^1^LSD post-hoc comparison revealed that P2s who were not helped reported *more annoyance* with partner’s decision than 1) conditionally helped P2s (MD: 2.54; p <.001), and 2) unconditionally helped P2s (MD: 3.01, p<.001). No other comparisons were significant.
^2^LSD post-hoc comparison revealed that ‘unhelped’ P2s reported *lower* gratitude than 1) conditionally helped P2s (MD: 4.21; p <.001), and 2) unconditionally helped P2s (MD: 4.88, p <.001). Meanwhile, conditionally helped P2s reported *lower* state *gratitude* than unconditionally helped P2s (MD: 0.675, p = .031).
^3^*All P2s* (N=12) who were not helped rated ‘1 (Not At All)’ for the item ‘I feel indebted to my partner’
^4^LSD comparison revealed that ‘unhelped’ P2s reported feeling *less indebted* to their P1 partners than 1) conditionally helped P2s (MD: 3.46; p <.001), and 2) unconditionally helped P2s (MD: 3.96, p <.001). No other comparisons were significant.
^5^LSD comparison revealed that ‘unhelped’ P2s reported *lower* eagerness to reciprocate than 1) conditionally helped P2s (MD: 2.79; p <.001), and 2) unconditionally helped P2s (MD: 3.61, p <.001).Conditionally helped P2s reported *lower eagerness to reciprocate* than unconditionally helped P2s (MD: 0.822, p =.026).
