# Supplementary material for: Gratefully Received, Gratefully Repaid: The Role of Perceived Fairness in Cooperative Interactions
Source: PLoS One. 2014 Dec 8;9(12):e114976. doi: 10.1371/journal.pone.0114976 (PMC4259482; doi:10.1371/journal.pone.0114976)

**Supporting Information 7: On-screen instructions for the game**

Example: A P1-P2 pair in *‘Low-cost’* condition with P1 making a *Conditional Offer* and P2 *Accepting* it

**I. Opening (All participants)**

Each of you is given 150 points at the beginning of this game. In this game there is a bonus threshold of 200 points, i.e. you will be entitled to a bonus (in addition to your £2-inconvenience allowance) only if you manage to hit 200 points (or above).

Each point above the threshold (200 points) is worth 1p in addition to the basic bonus £2. For example, if your final score is 300 points, the total bonus for you will be:
Basic bonus for reaching 200 points: £2 + Extra bonus: (300-200)* 1p= £ 1🡪Total £ 3
The maximum points you can get is 350, meaning that you could at most end up with a bonus of £3.50 (£2-basic bonus+150*1p)

In this game you will be asked to roll a fair die once, and afterwards enter the number you get to the computer. The system will then generate a CHANGE in your score based on the number you get out of the die-rolling. Dependent on your die-rolling results, there are SIX possible changes could be brought to your final scores, namely: i) -50, ii) Zero change, iii) +50, iv) +100, v) +150, vi) +200.

Please note that a large number rolled does not necessarily mean a more favourable change and vice versa. Instead, the level of your final bonus is jointly determined by WHAT YOU AND THE OTHER PARTICIPANTS HAVE GOT; in a way that the RARER number you get relative to others, the MORE FAVOURABLE change in your score. For instance, If there are 20 players and you roll ‘1’ and five others roll ‘2’, another five people roll ‘3’, four people get a ‘5’ and five others roll ‘6’, you’ll get the most bonus because you had the rarest number.

In this game, everyone has a partner. Nevertheless, neither joint effort nor competition with your partner will be required to capture the bonus. You and your partner are NOT IDENTIFIABLE to each other and will remain anonymous. The payoff of yours and your partner’s will be shown on-screen shortly after you key in the number you get after rolling the die.

Please note that in this gaming session partners interact with one another and they will at times take turn to make decisions. As a result, you will see a couple of waiting screens while your partner is making his/her decision. A few waiting screens could last for 5-6 minutes, depending on how many decisions your partner is asked to make. Similarly, while you are asked to make a series of decisions, it will be your partners' turn to be shown the waiting screen

**II. Die Rolling and Entry of results (All Participants)**
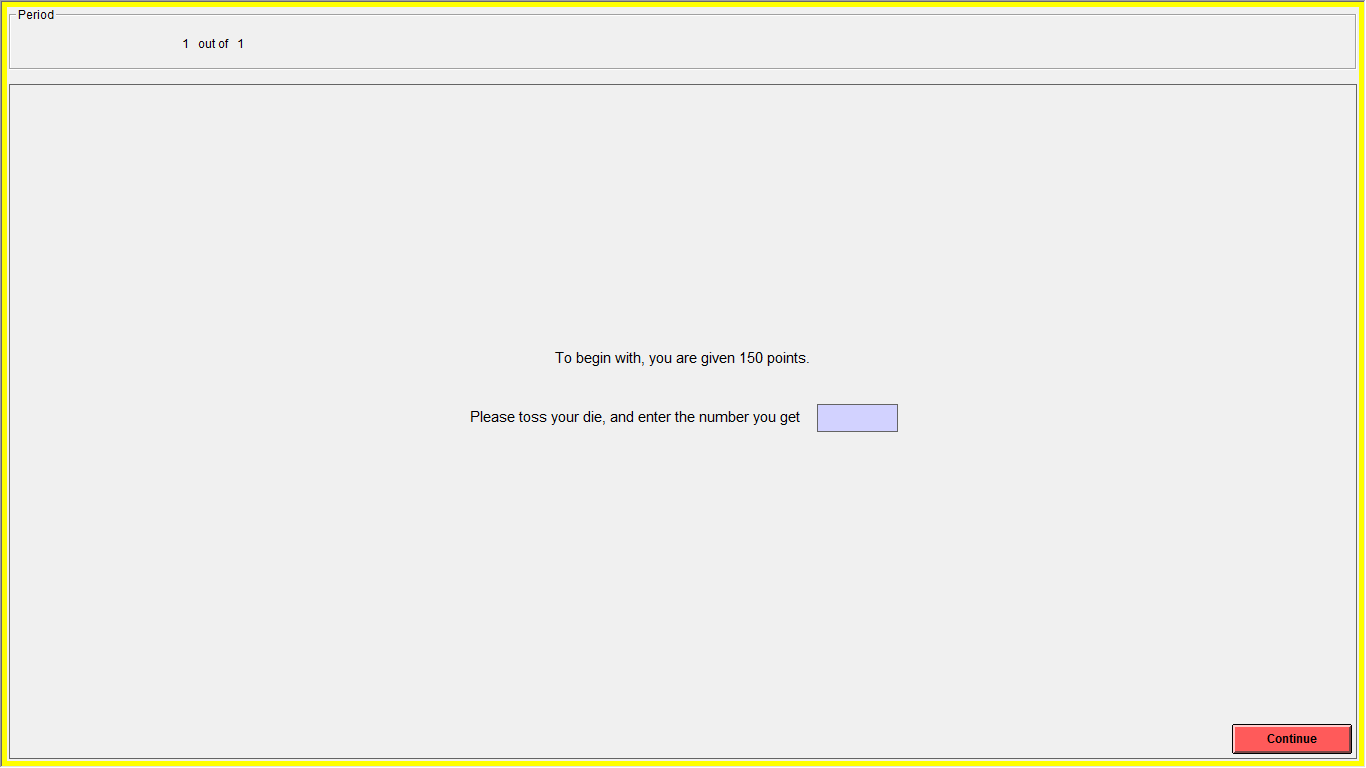


**III. Display of INITIAL payoffs of players and that of their partners (Player 1s +Player 2s)***Player 1s* : **Own** Payoff Display
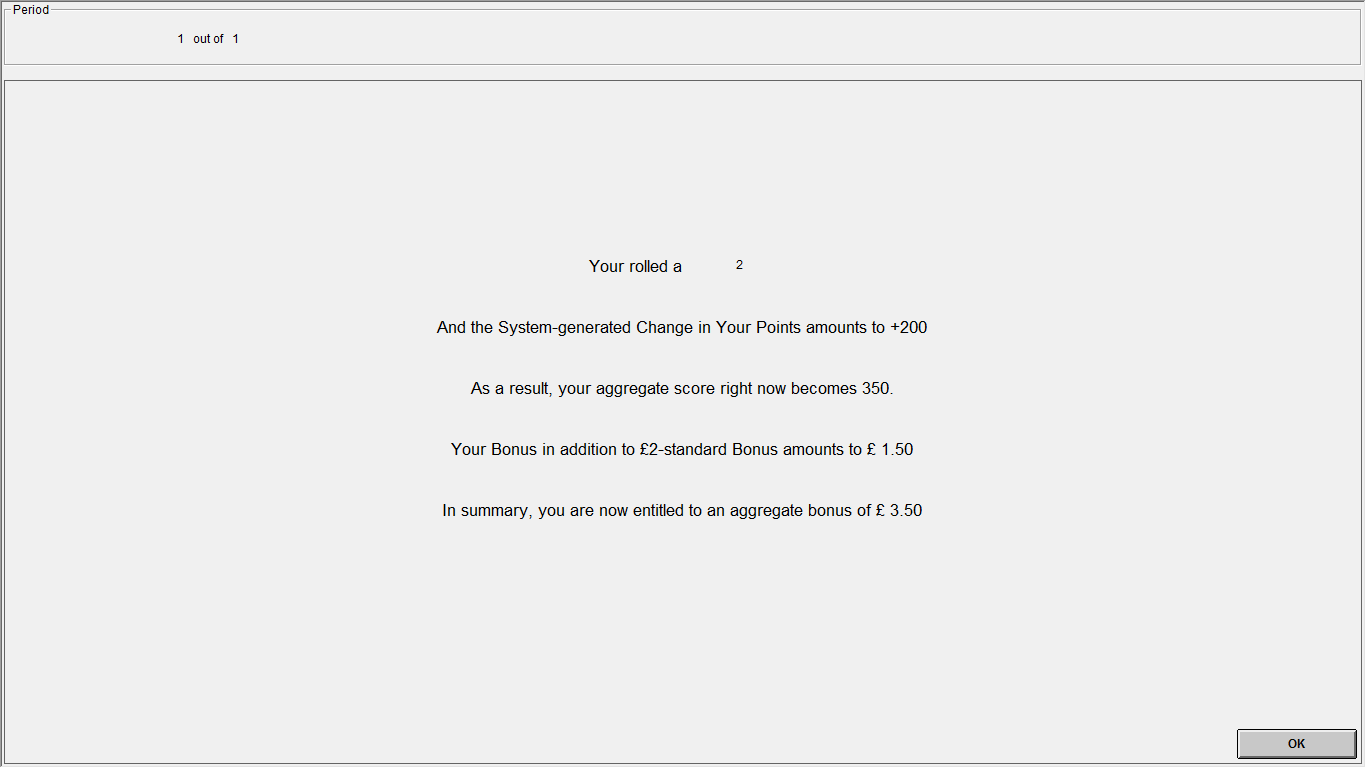


*Player 2s* : **Own** Payoff Display
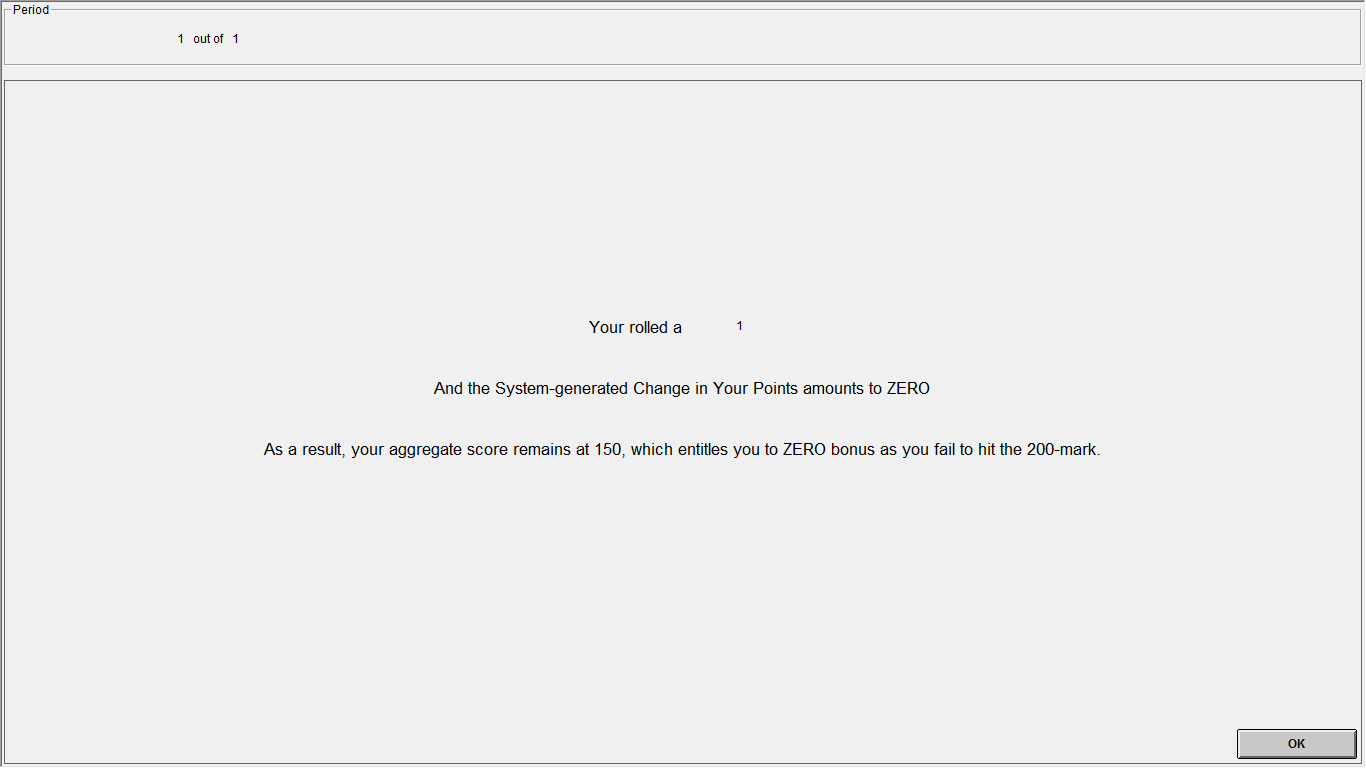


*Player 1s*: **Partners’** Die-rolling Score (and Payoff) Display
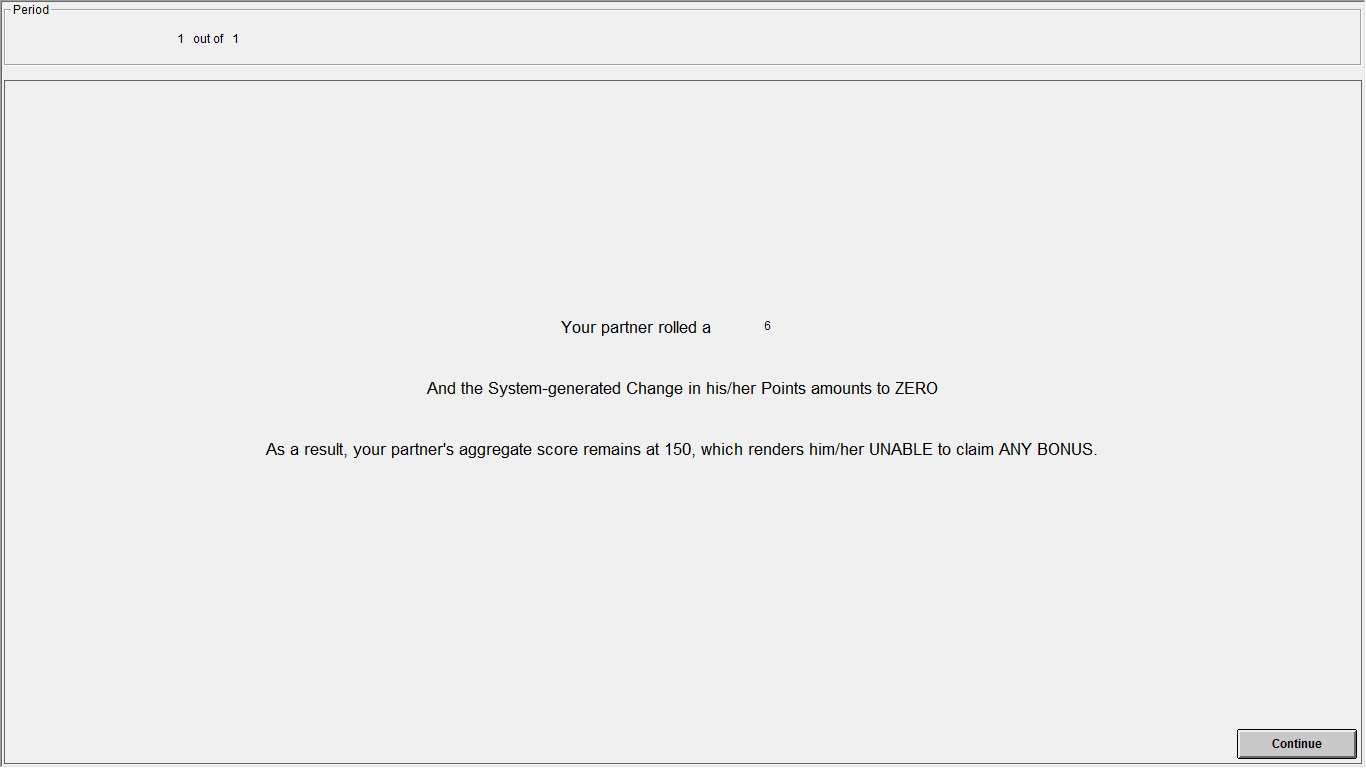


*Player 2s*: **Partners’** Die-rolling Score (and Payoff) Display
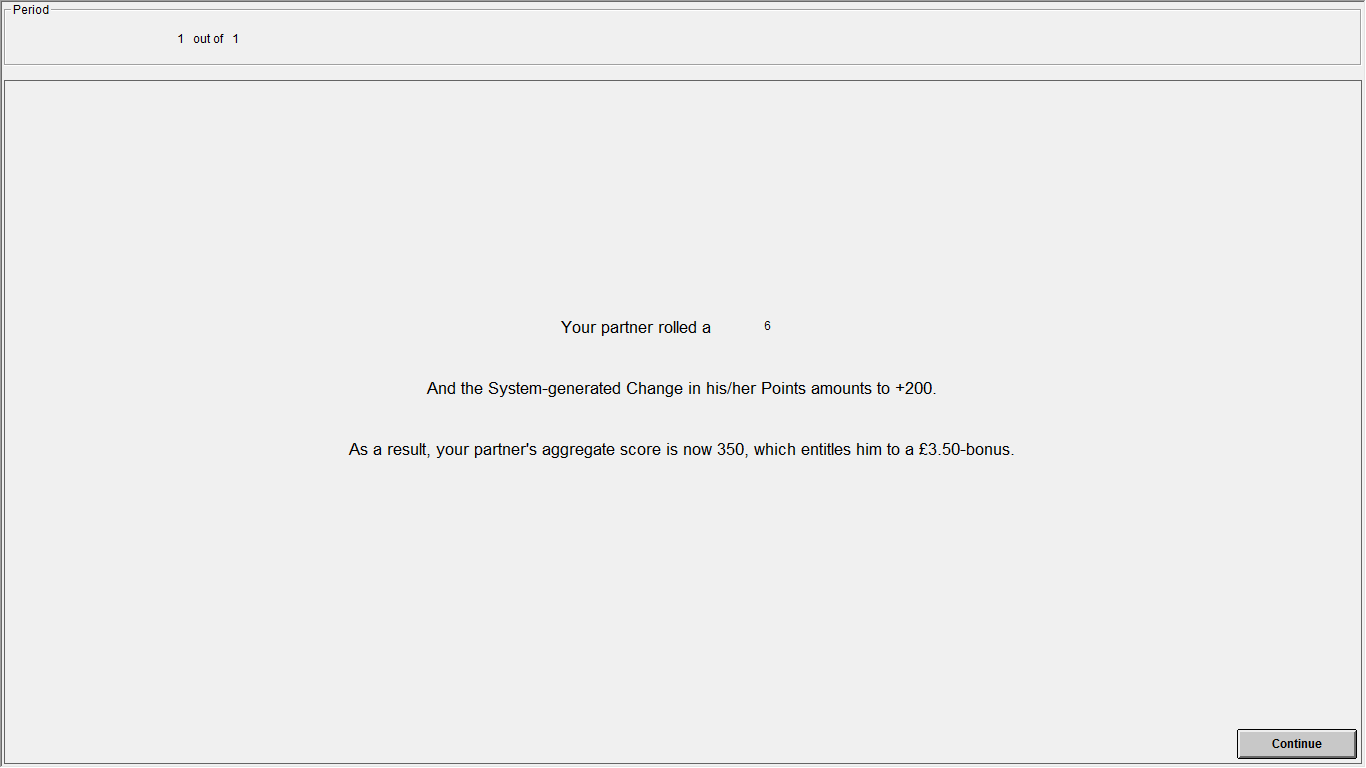


**IV. Pre-offer Ratings: Attributions of partners’ Die-rolling** (e.g. Player 2s)
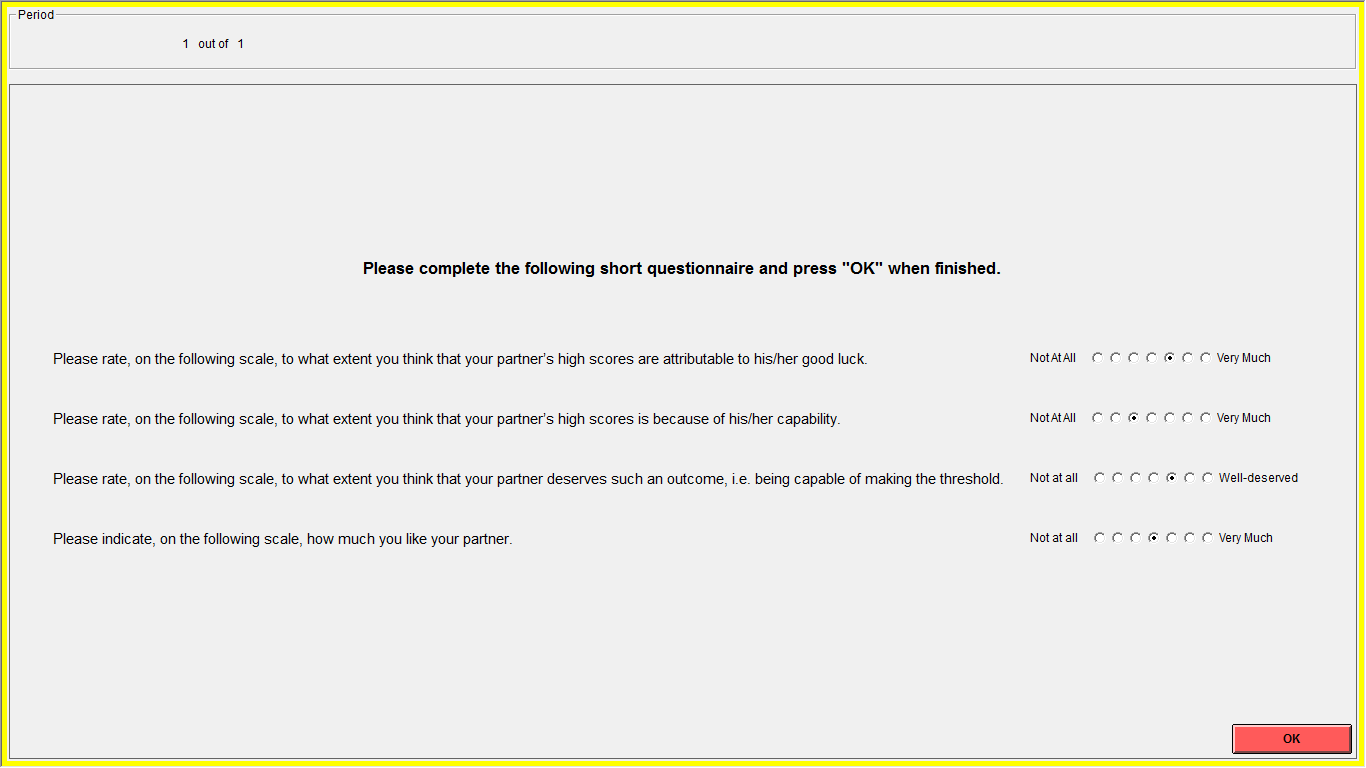


**V. Waiting Screen (Player 2s) and Helping decisions (Player 1s)**a. Player 2s’ Waiting Screen (while P1s made helping decisions)
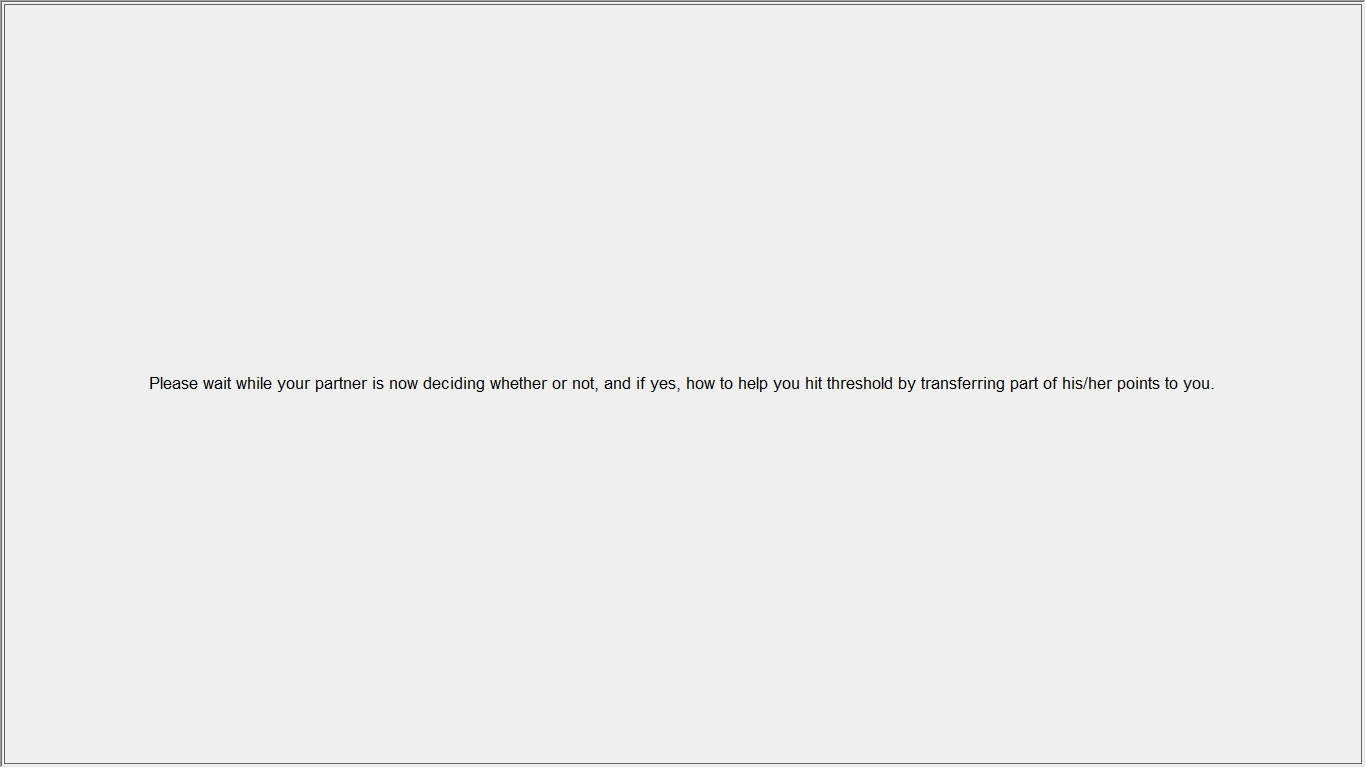
b. Helping Decisions by Player 1s
bi. Rules of Making an offer (Player 1s)
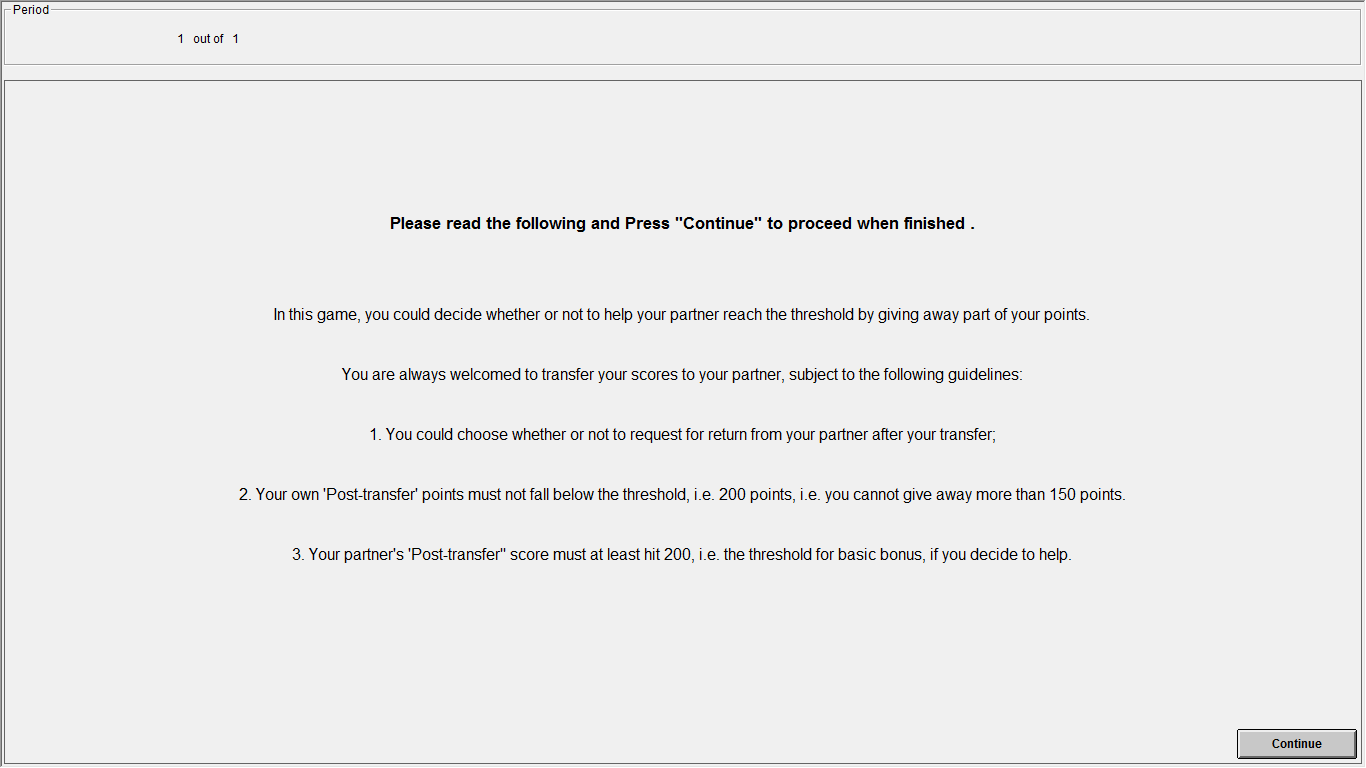


bii. Understanding Check for the Rules of Transfer
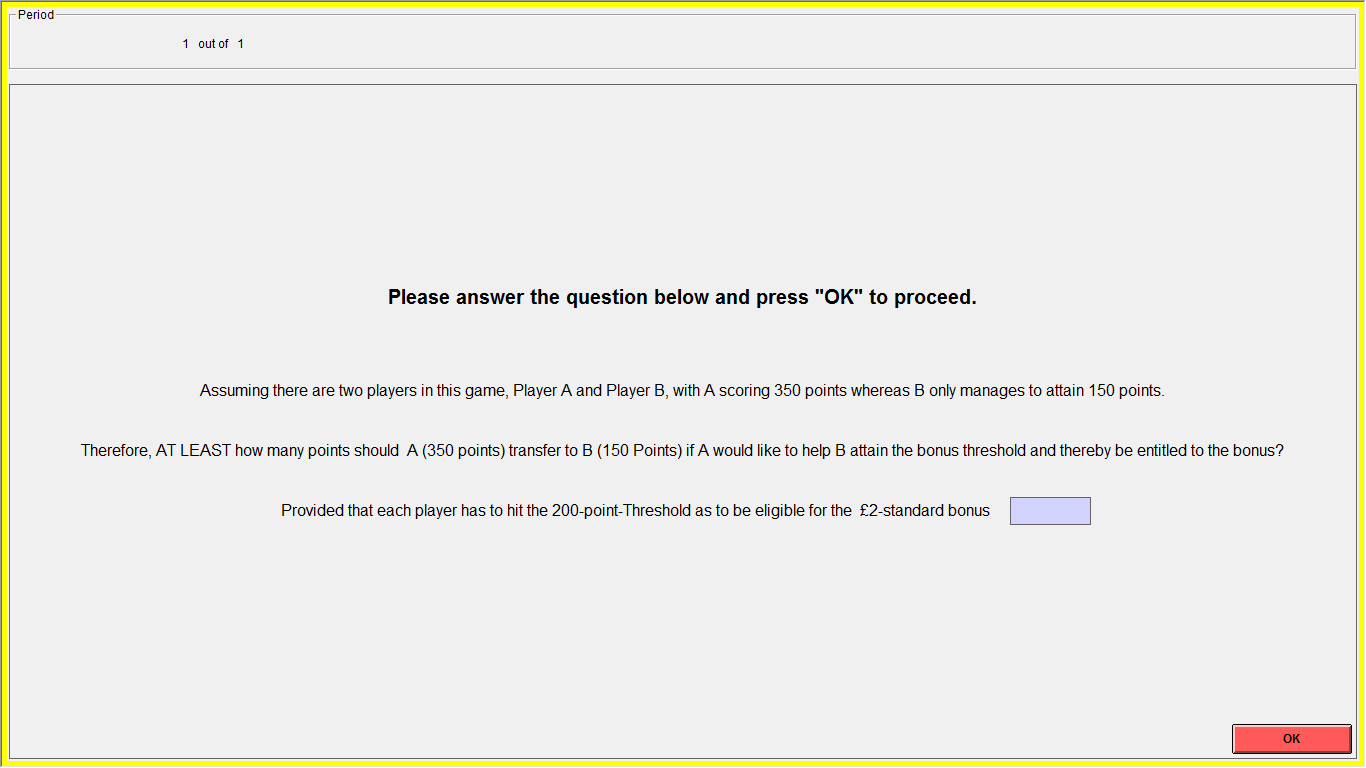


biii. Player 1s’ Decision to Make an Offer (I to III)
I) Basic Ground Rules and Notification of Partners’ ‘Need for Help’
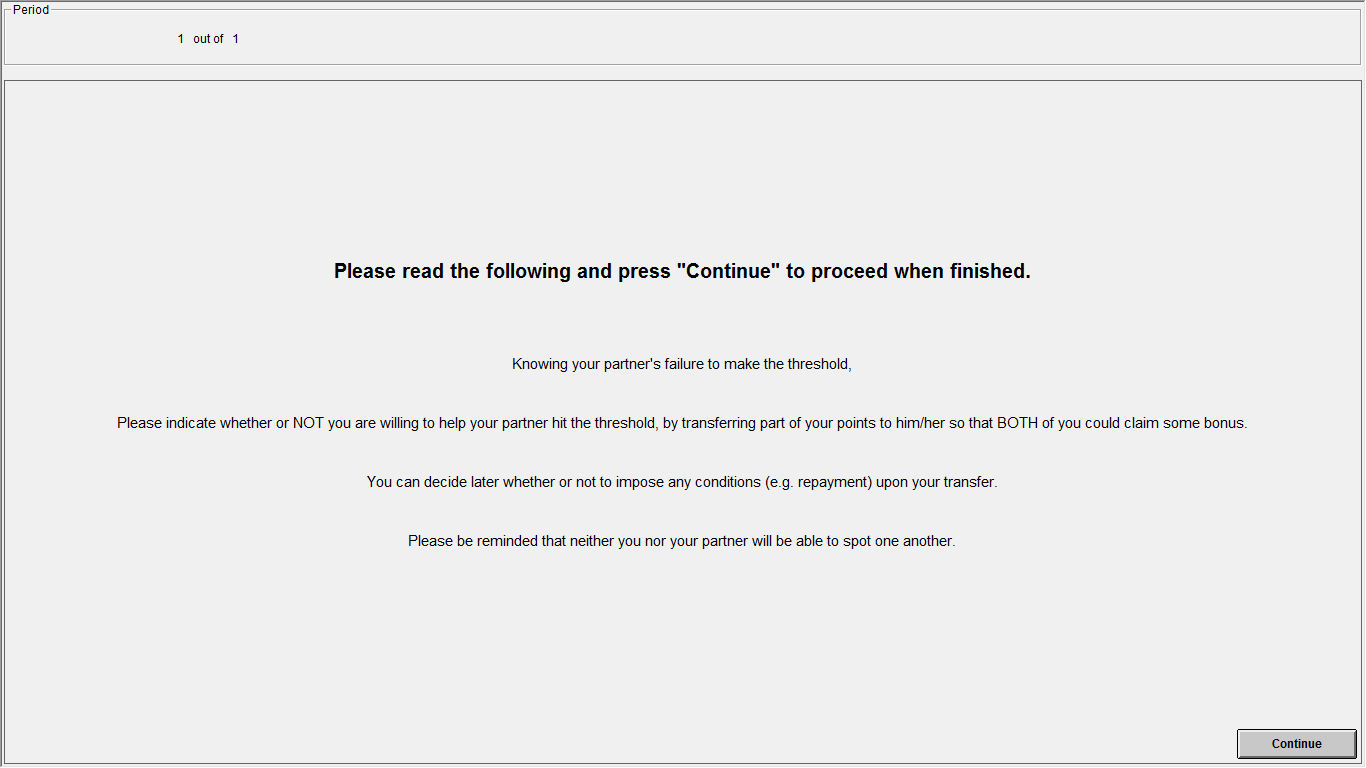
II) **To Help or Not** to Help
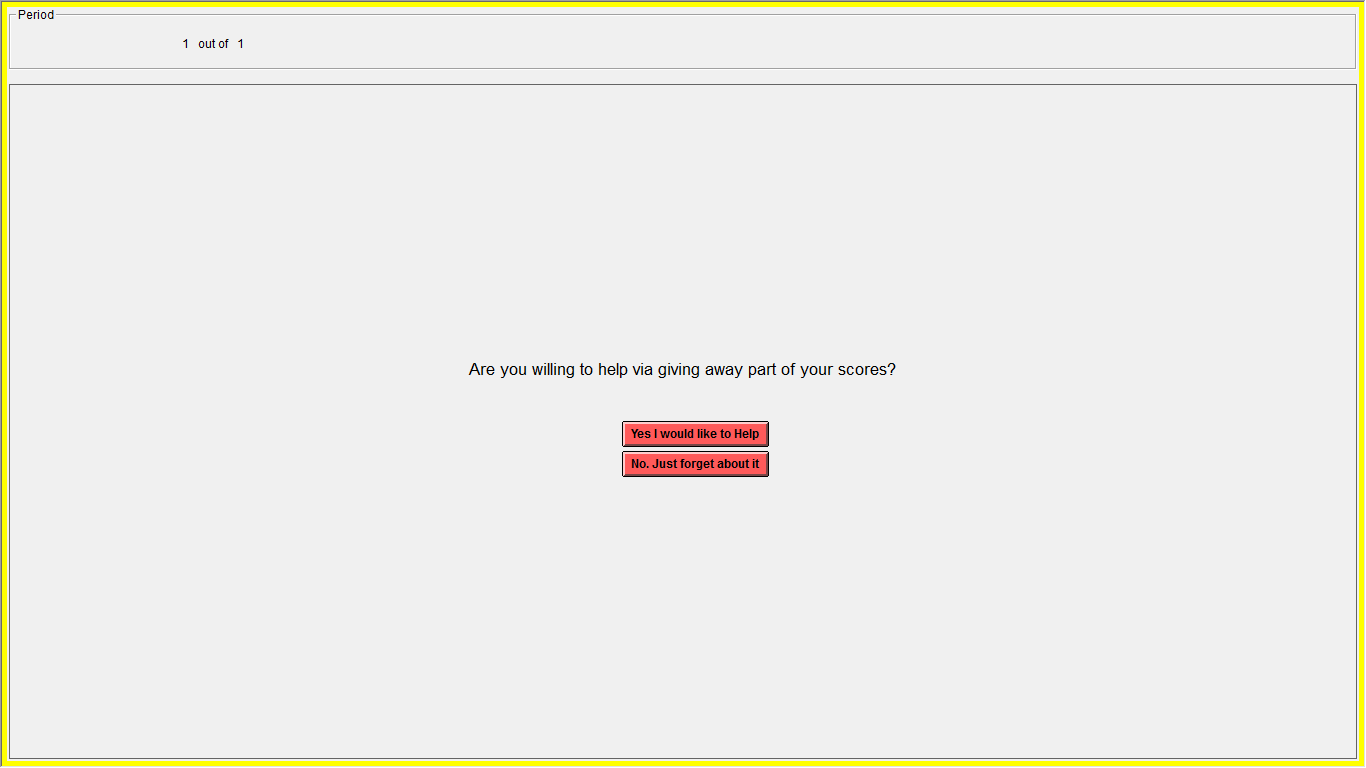


III) **Conditionality** of Offer: Whether to impose a *‘repayment clause’*?
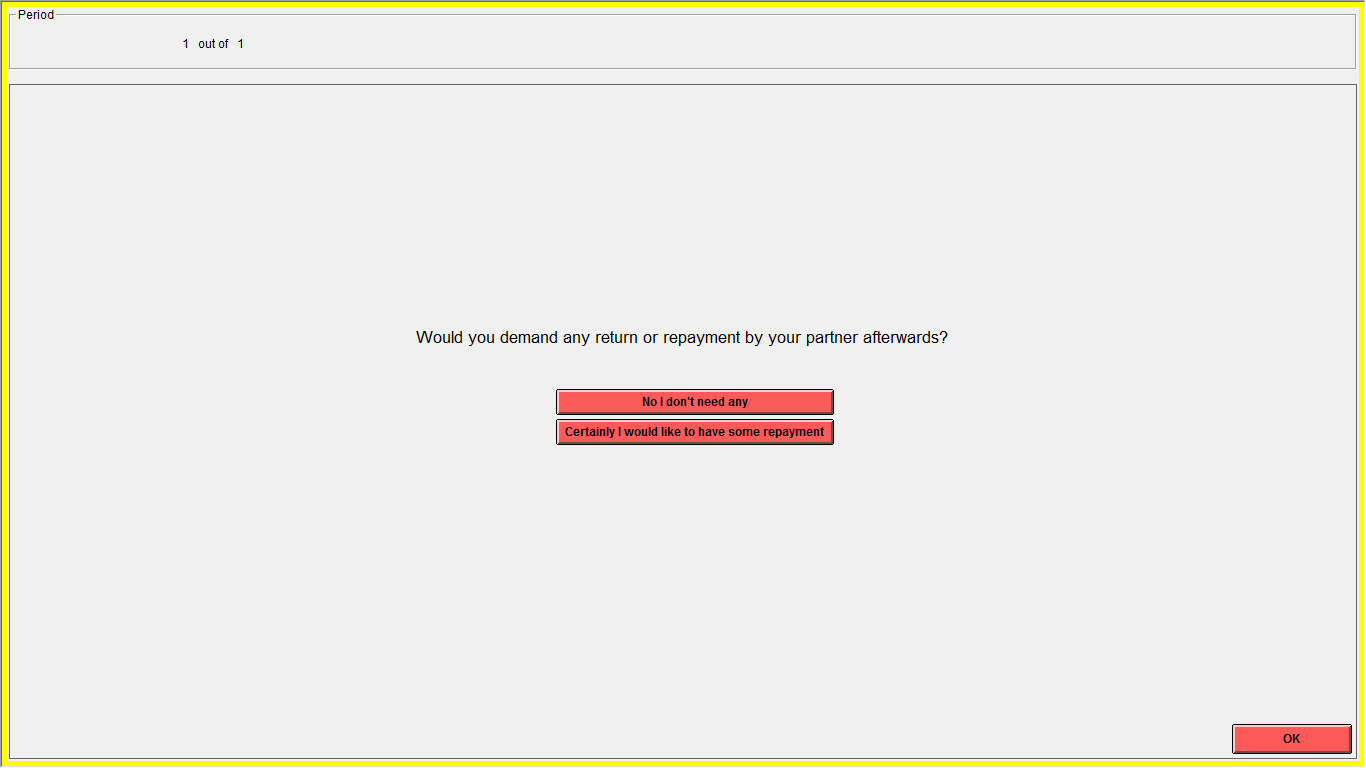


biv. Player 1 after agreeing to make a Conditional Offer (I TO III)
(I) The **list of Available** ‘Repayment Clauses’

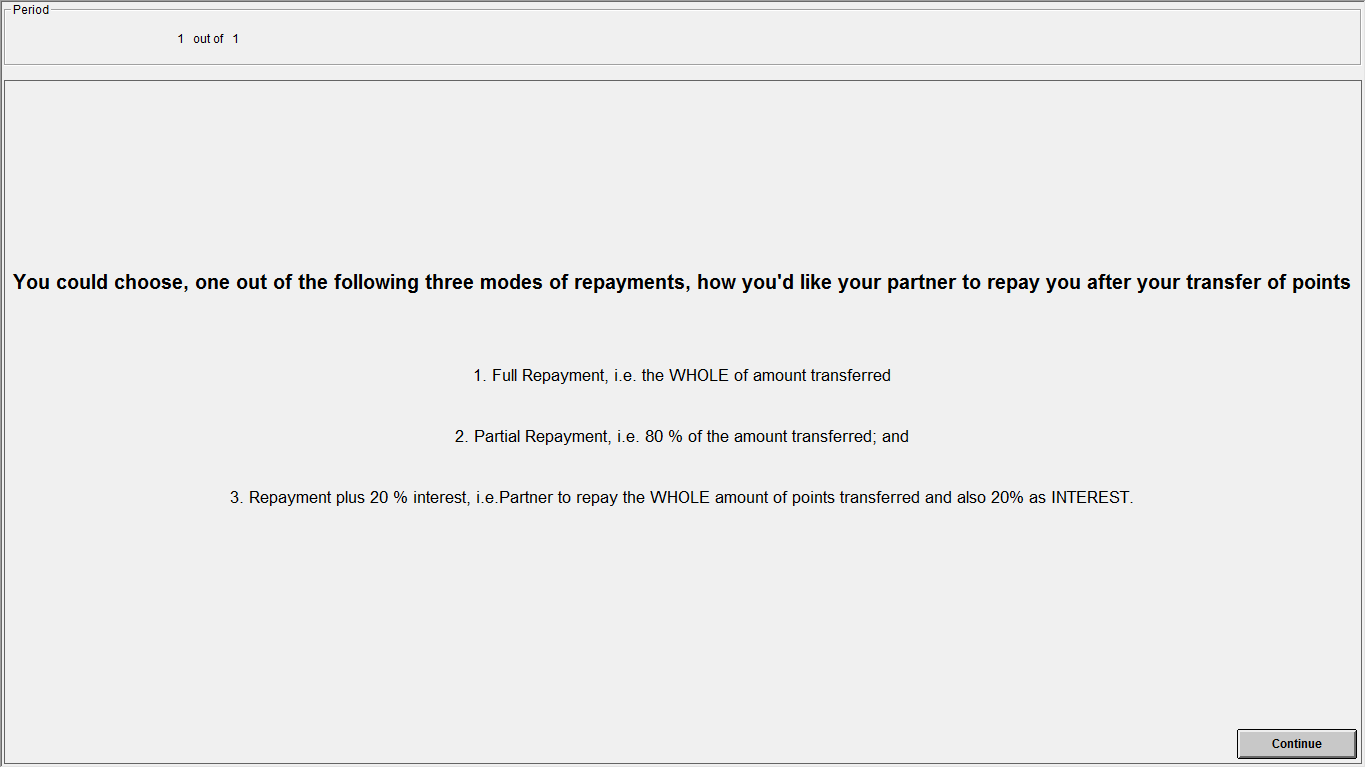
(II) Picking a ‘Repayment Clause’ : Full vs. Partial Repayment vs. Repayment Plus Interest (20%)
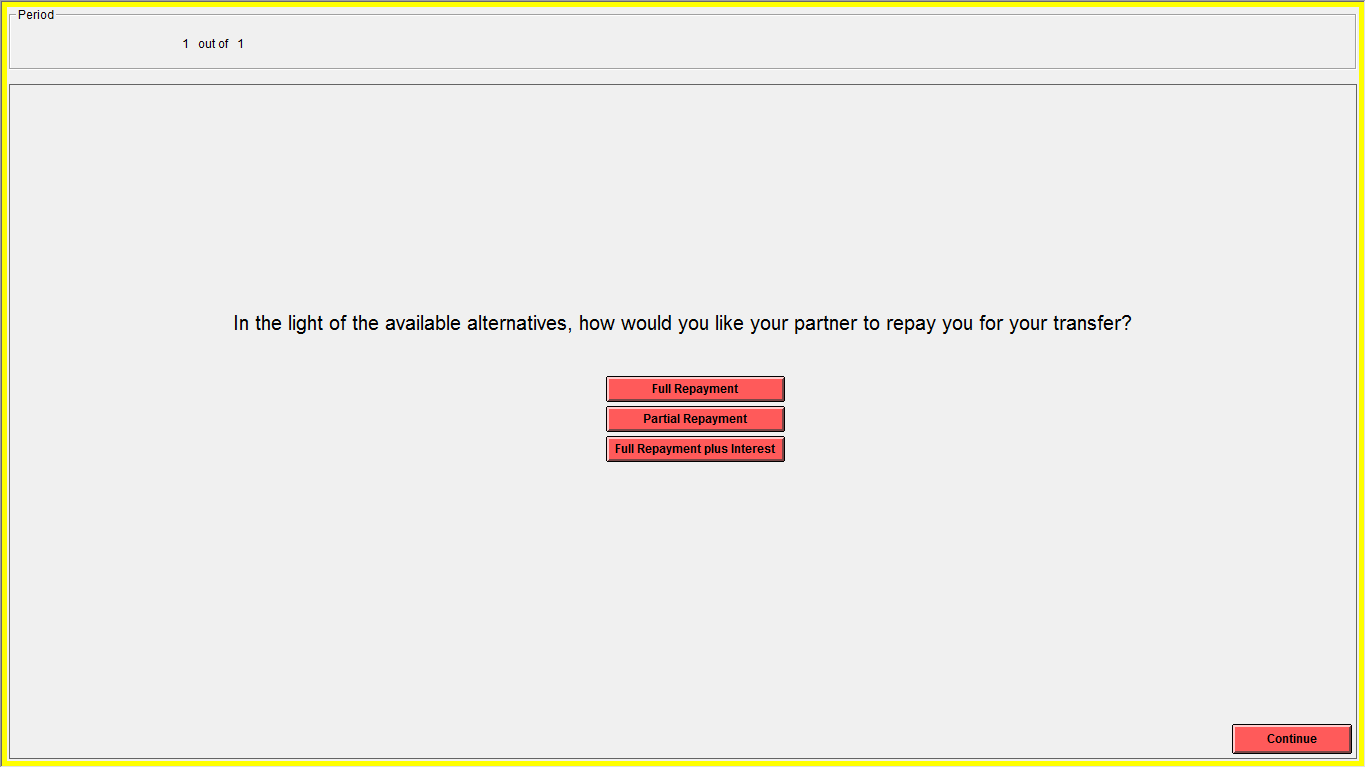


(III) A Reminder: Rules of Making a Transfer
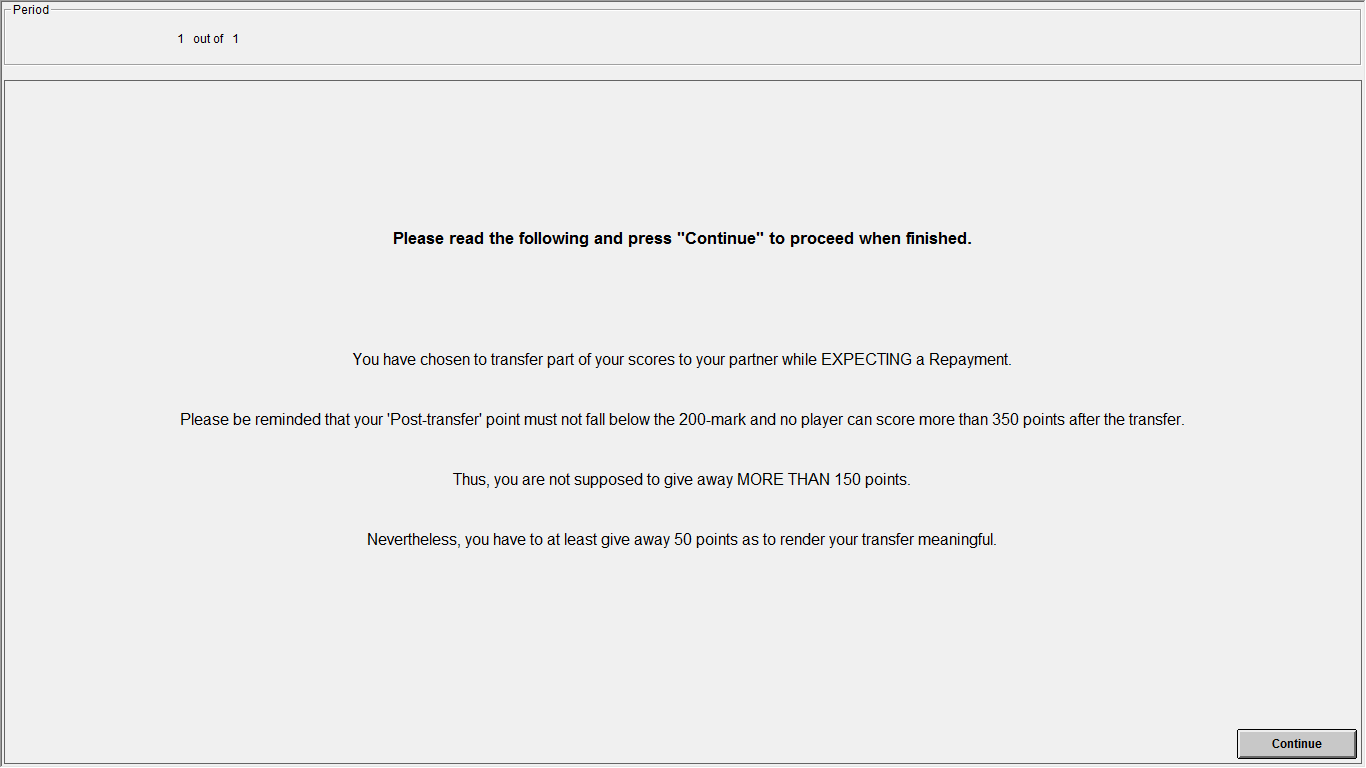
(IV) Deciding the *Magnitude* of Transfer
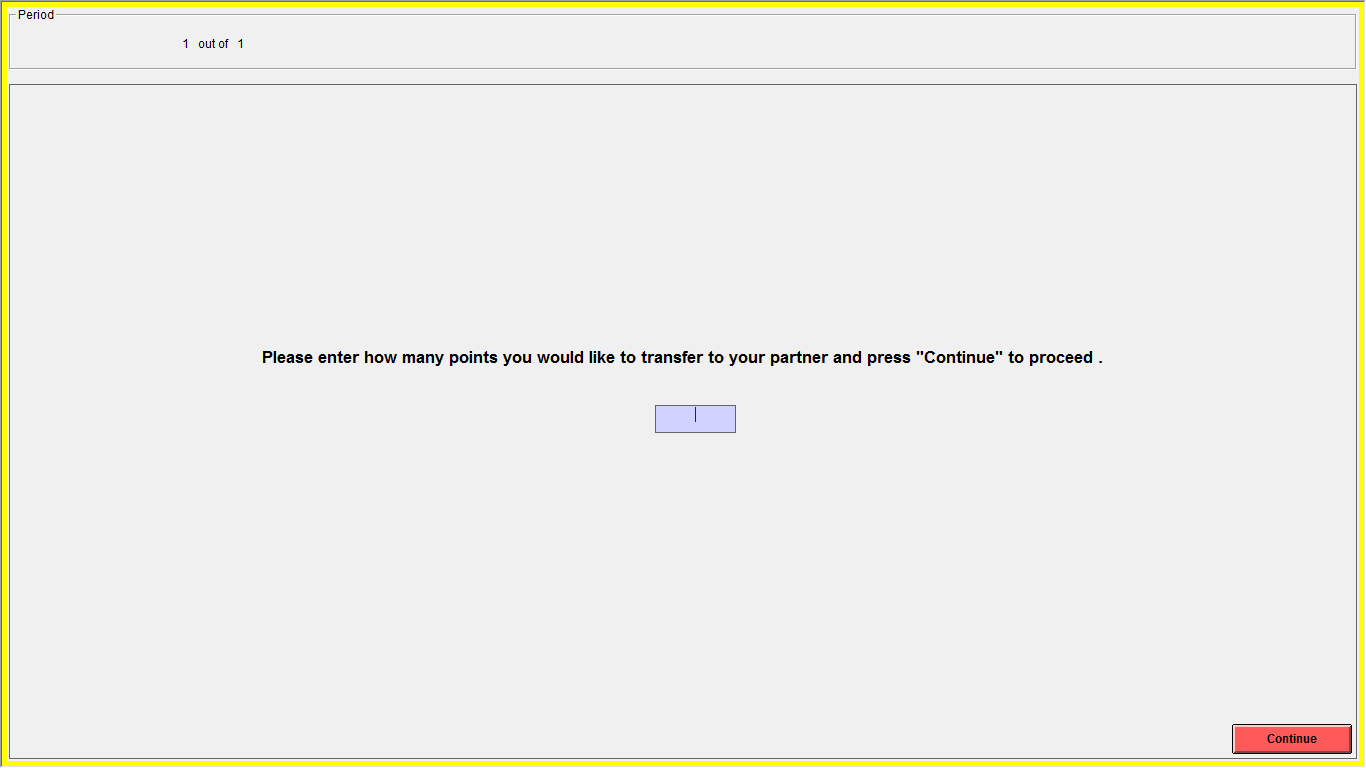


**V. Offer Acceptance, Post-offer Ratings, and Repayment for Player 2s**a. Waiting Screen for Player 1s (while P2s made their decisions as regards their offers received)
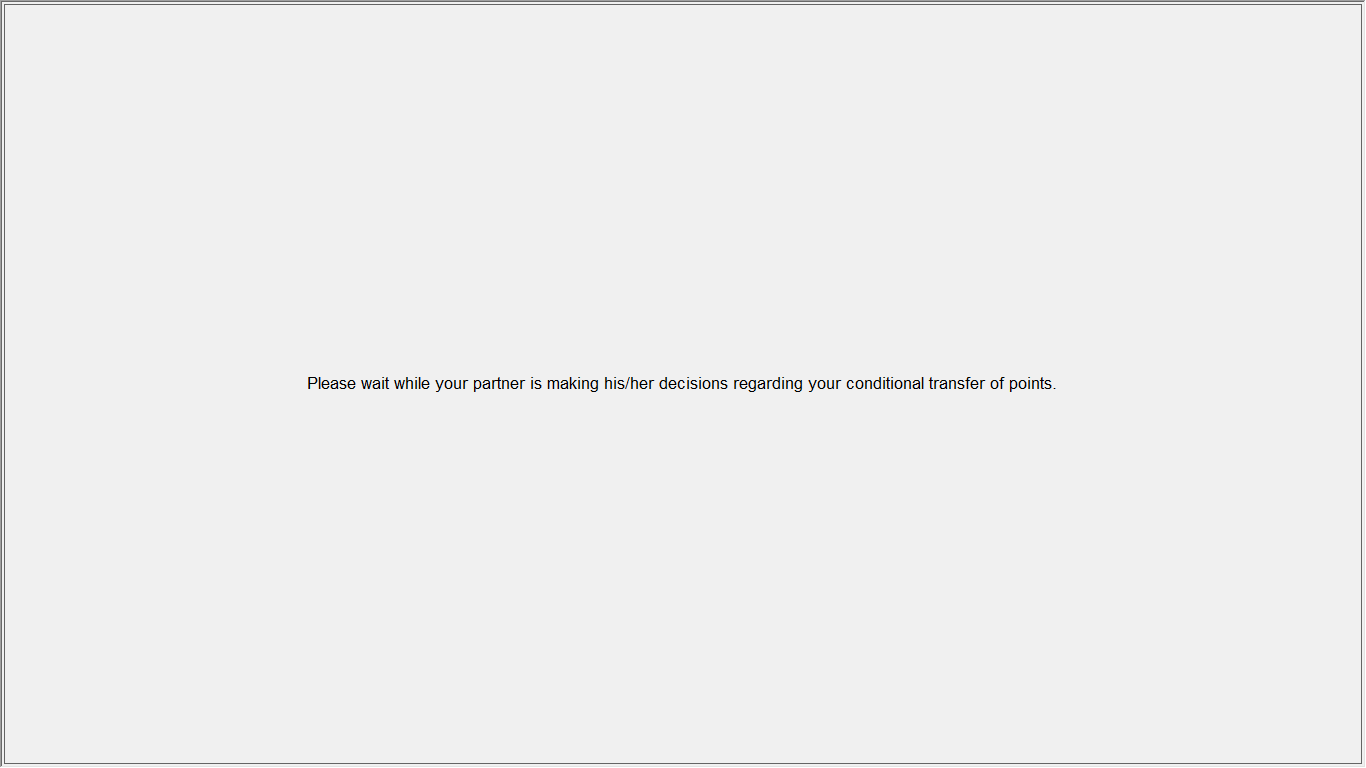
bi. Offer Display and Acceptance (or Rejection) for Player 2s (I to IV)
(I) Partners’ Decisions (*To Help or not to have helped*)

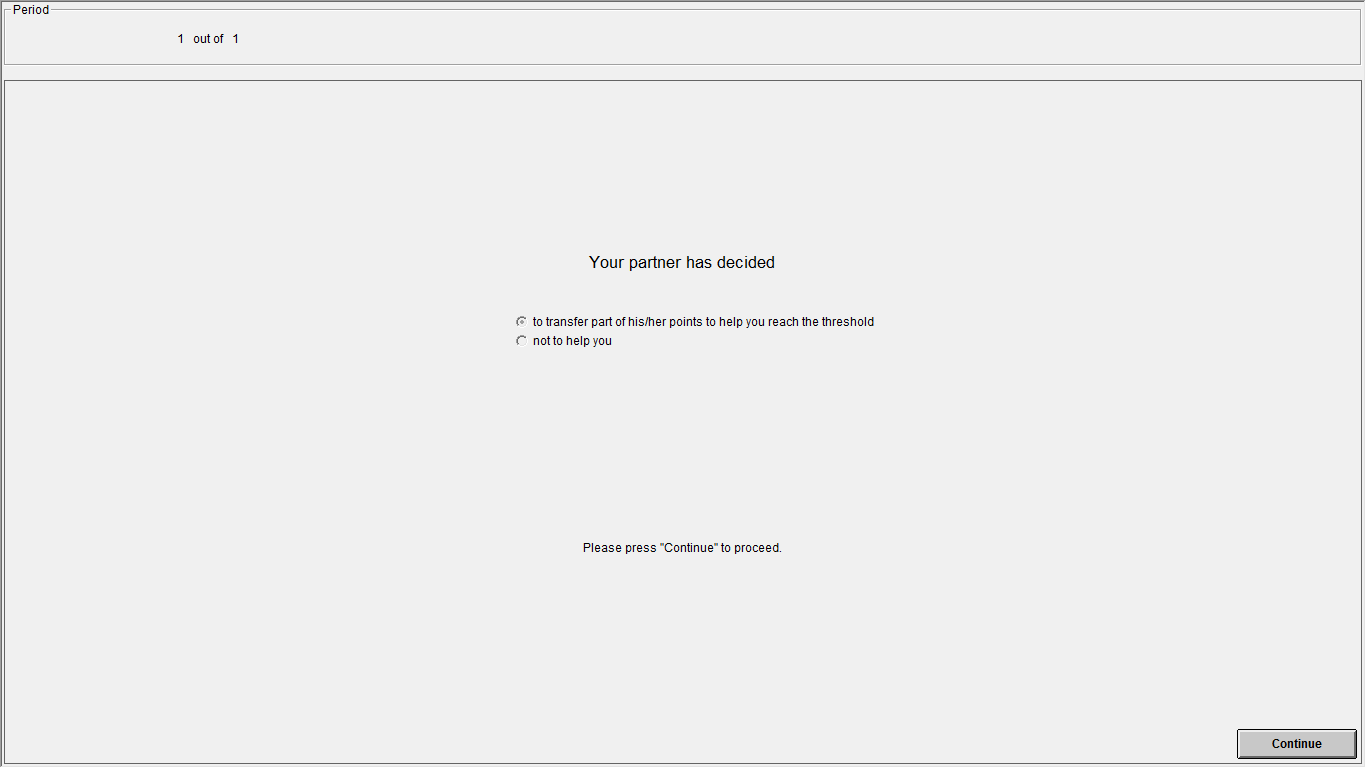


(II) Understanding Check
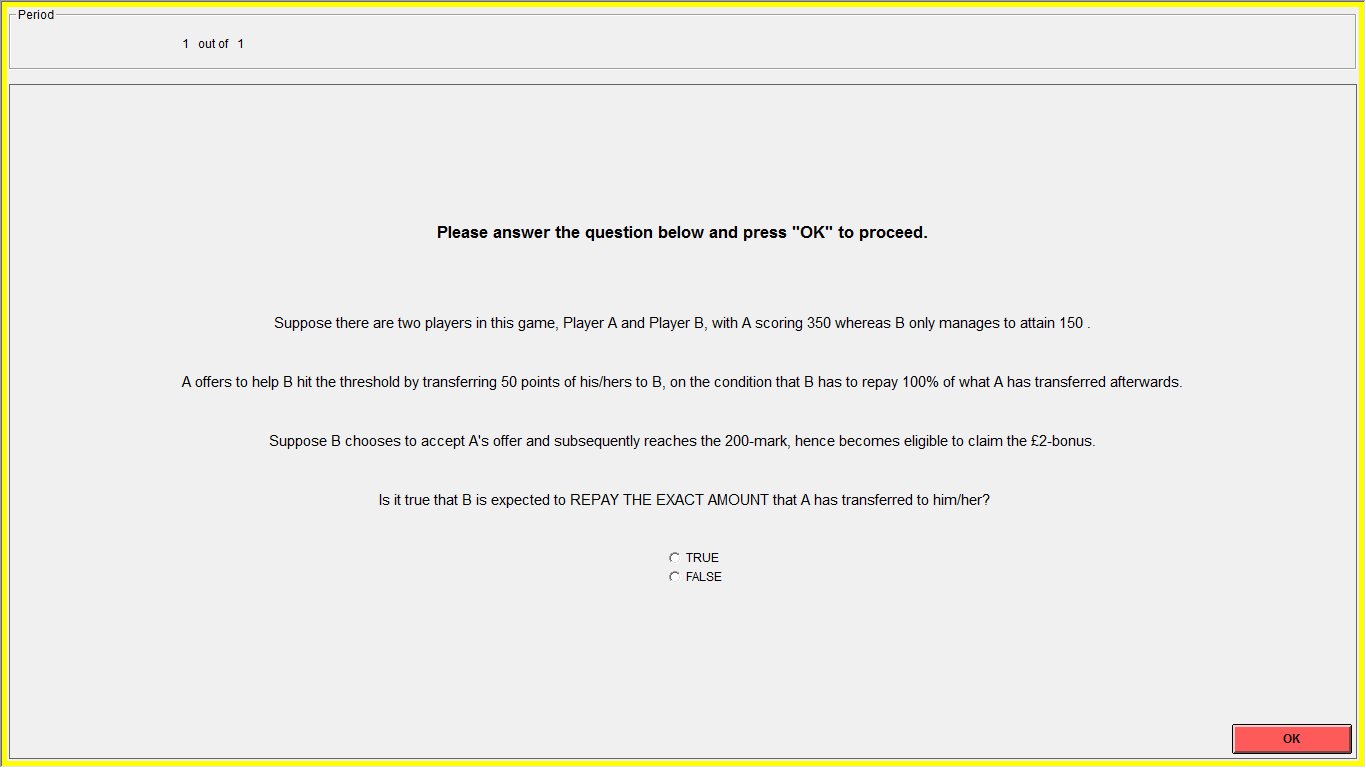
(III) Offer Display
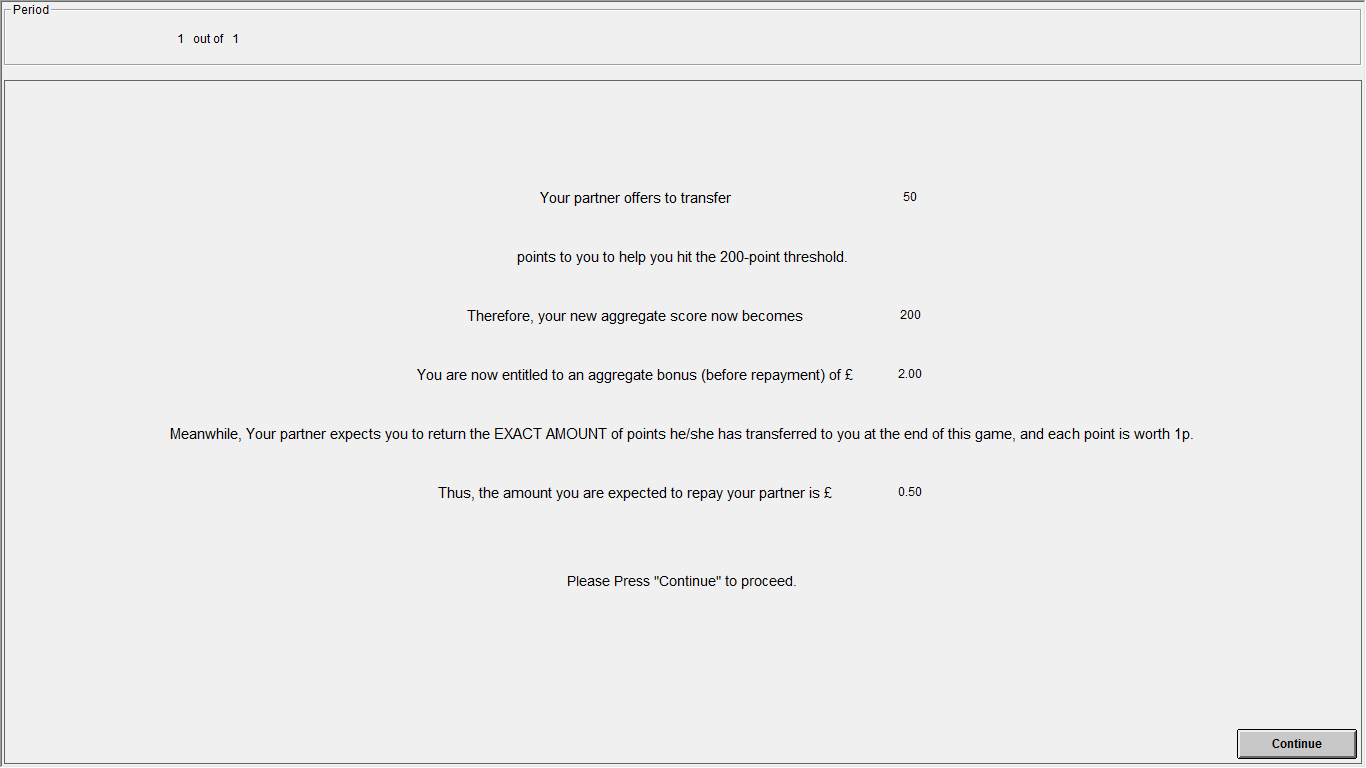


(IV) **Acceptance** or **Rejection** of the Offer
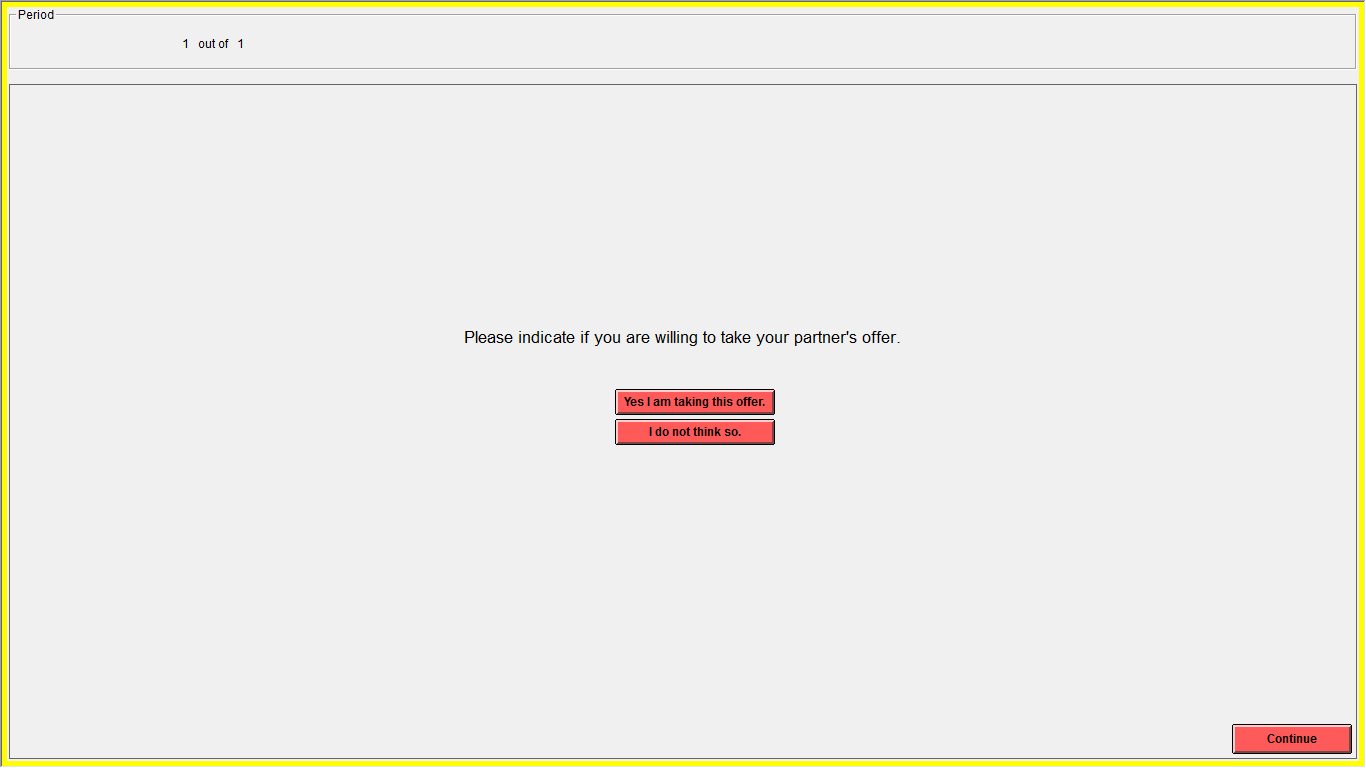
bii. Post-offer Ratings by Player 2s
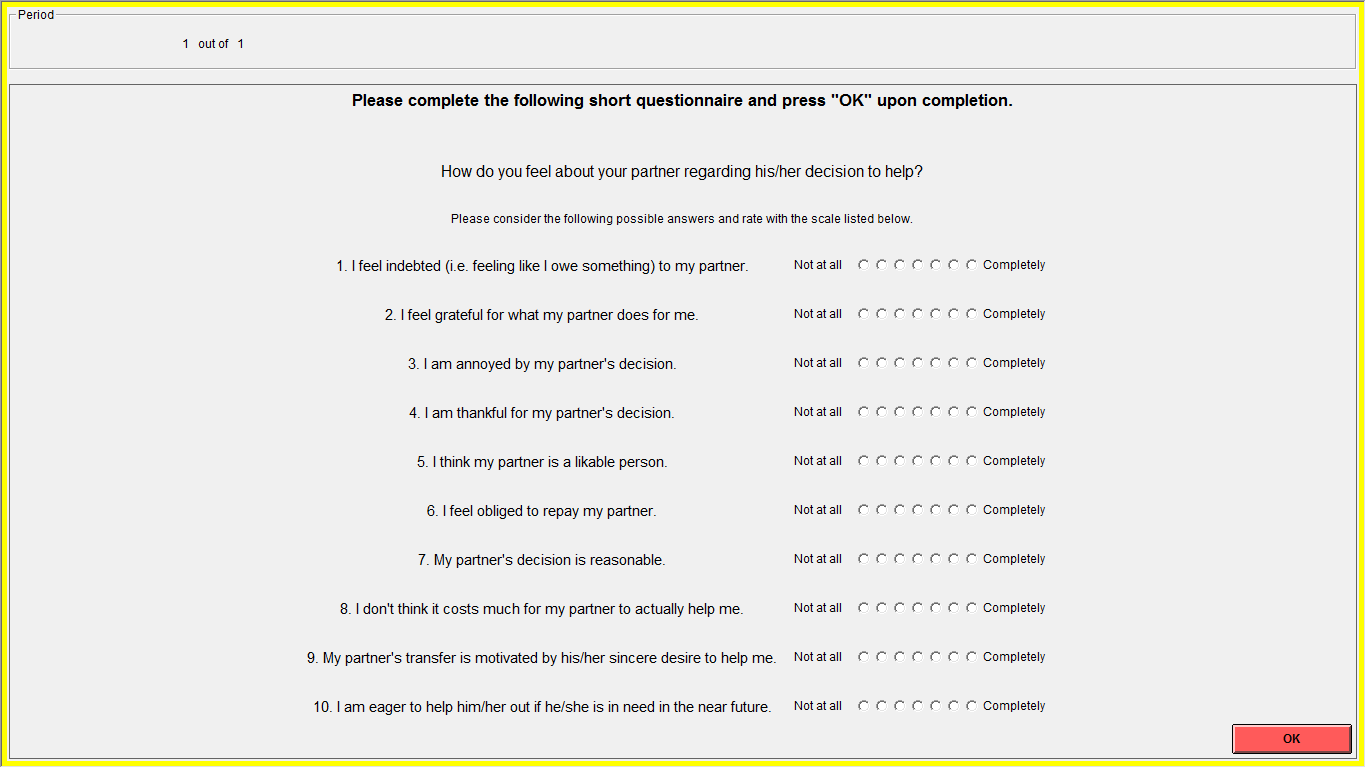


biii. Repayment Screens for Player 2s who **Accepted** their *Conditional* Offers (I to II)
I. A Reminder: ‘Terms’ of the Repayment
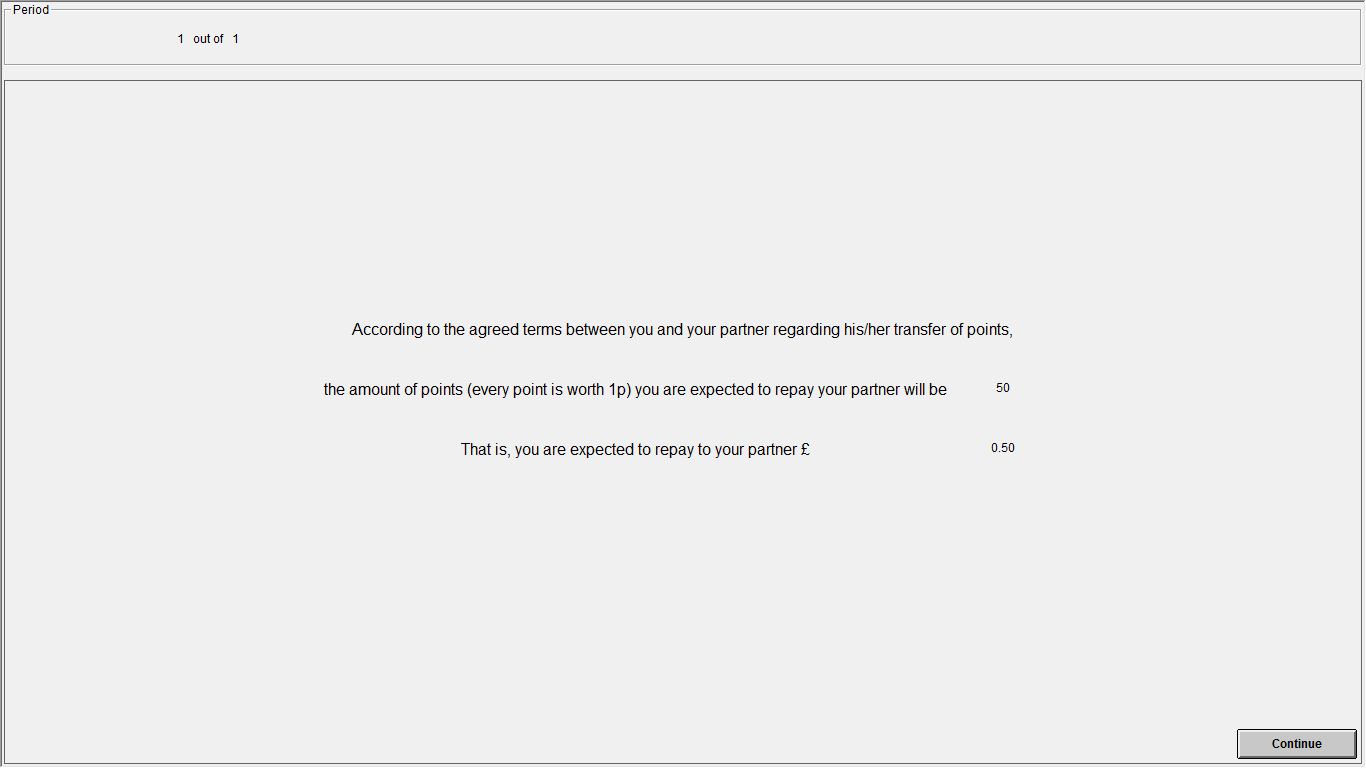
II. Repayment Decisions: P2s to **Freely decide** their *magnitude of repayment*
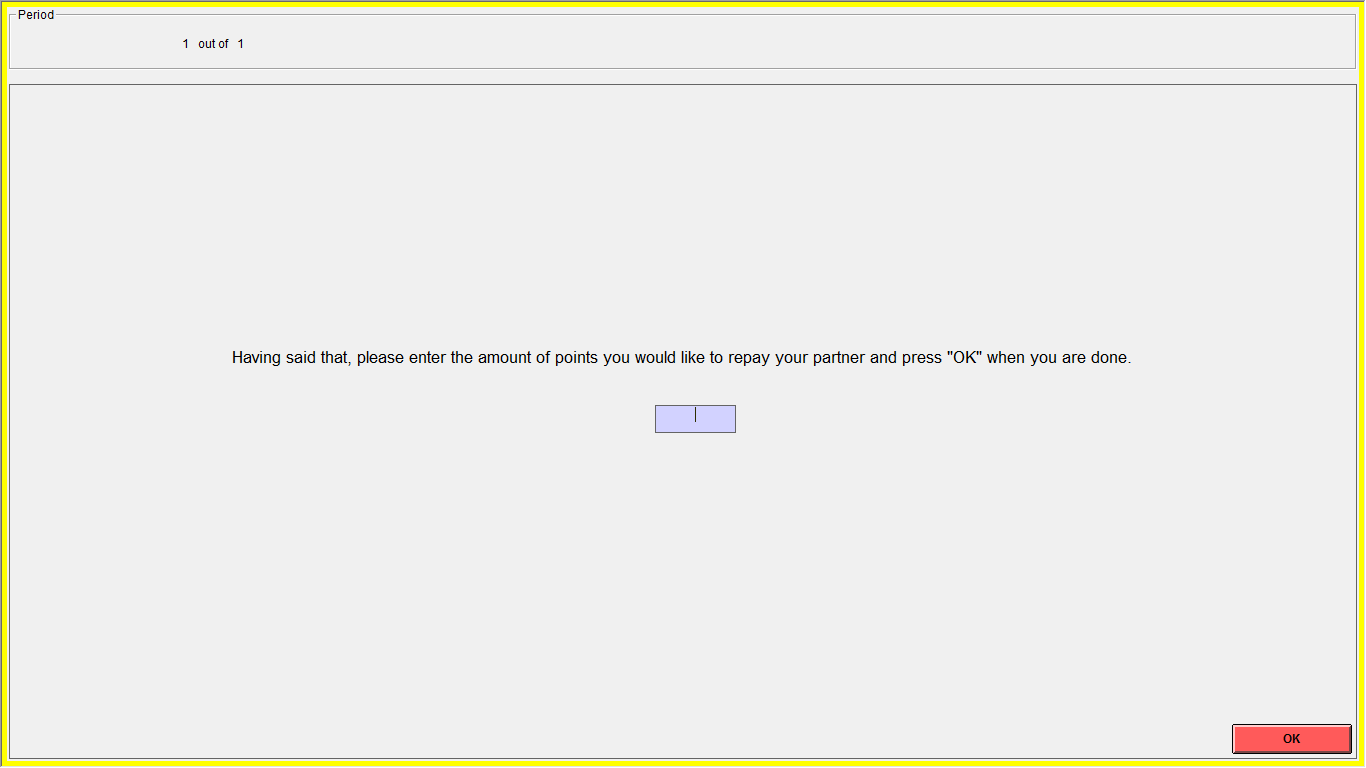


**VI. Compensations Display**Player 1s- CONDITIONAL Helpers whose recipients *did not defect* in this example
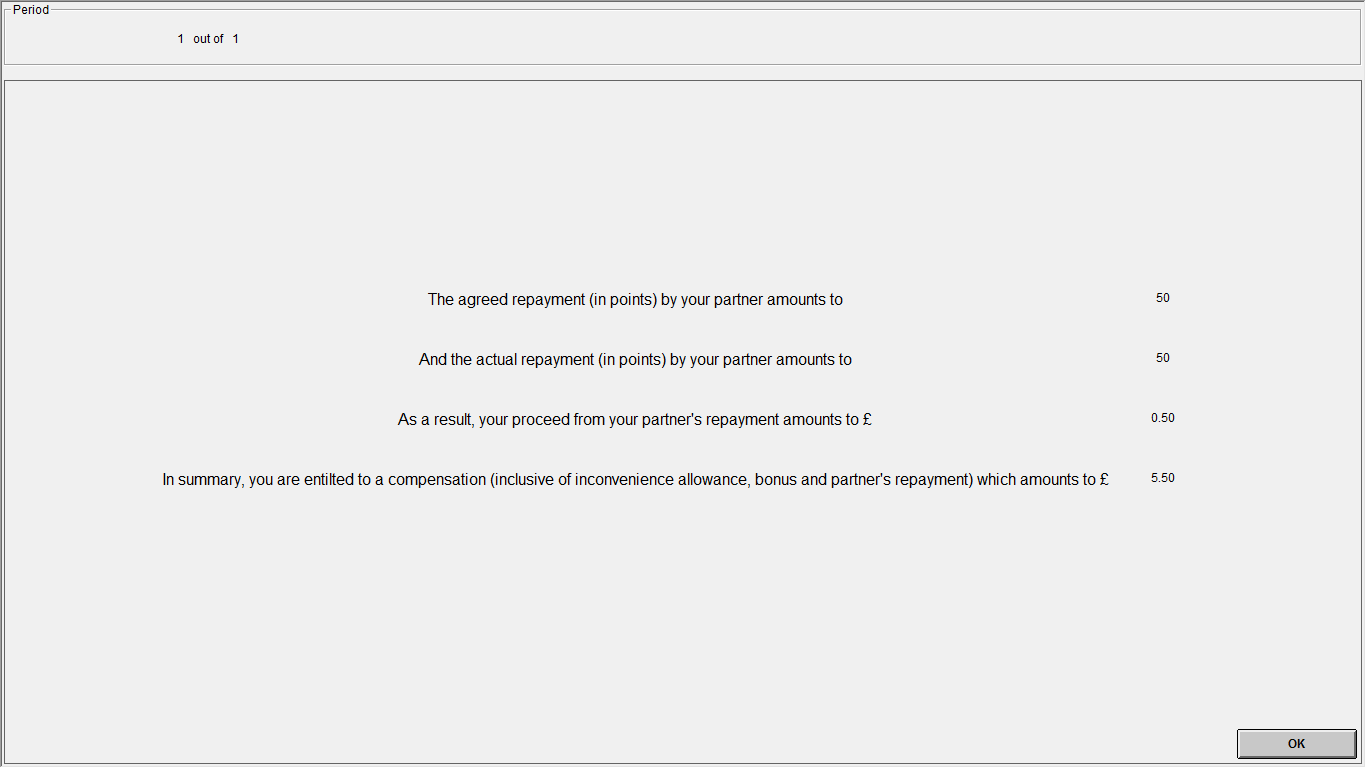
Player 2s - Recipients of *Conditional* offers and **Repaid Fully** (i.e. *NO* ‘breaching of contract’)
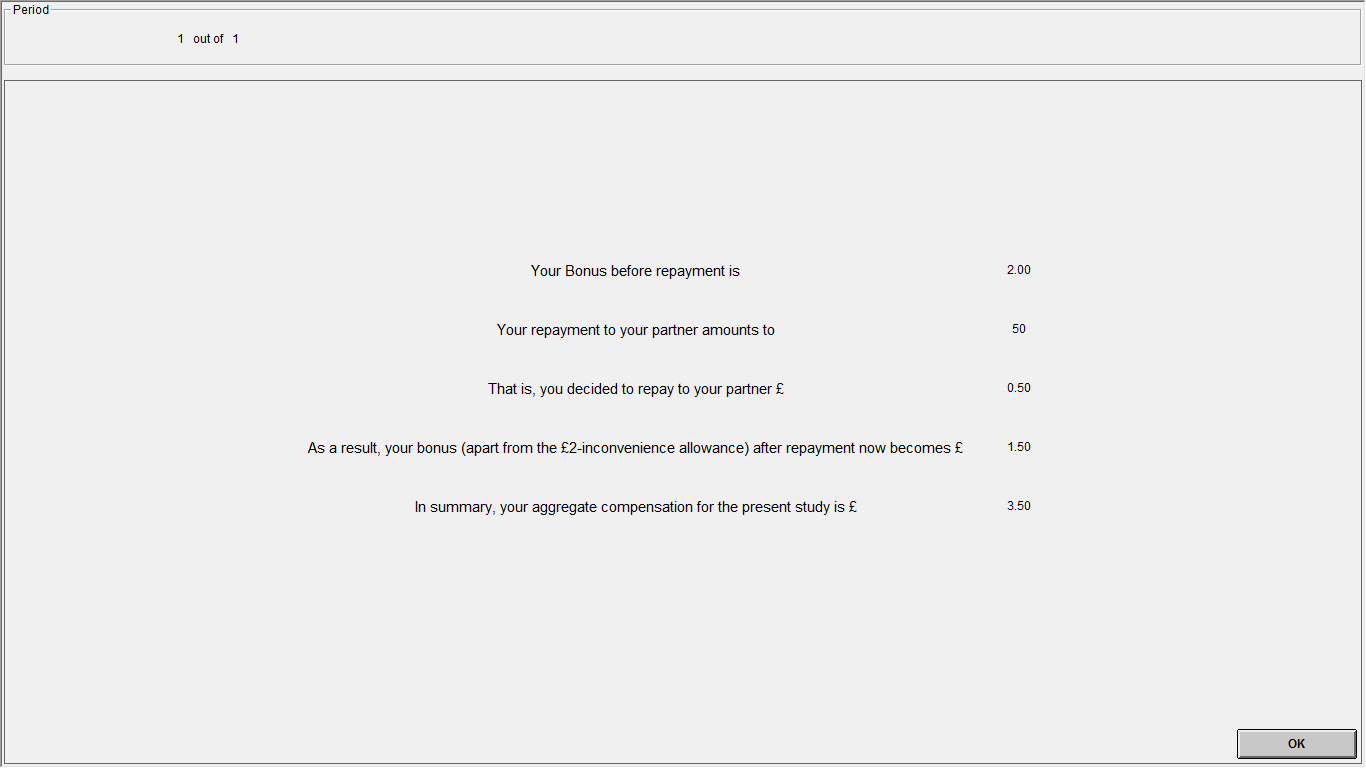


**VII. The End of the Game (All participants)**


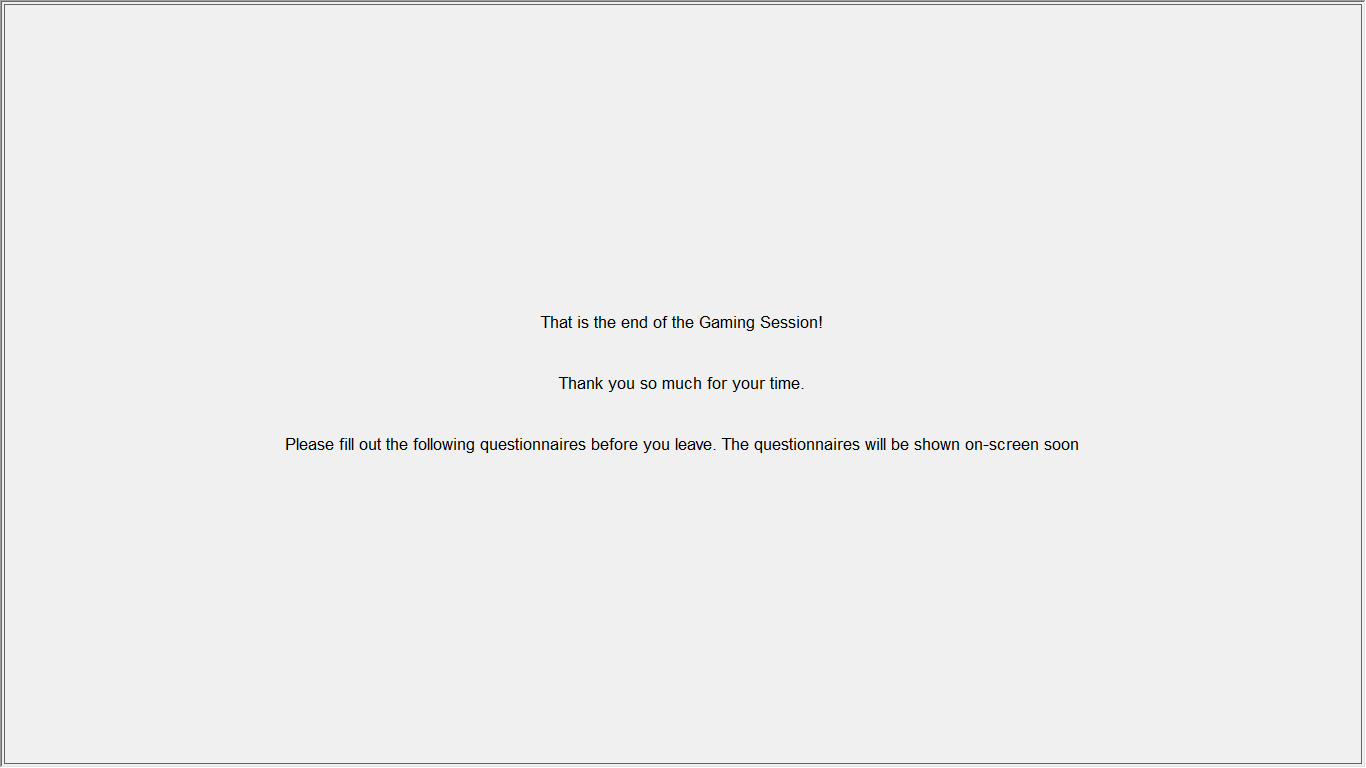

Supplement: S7 Supporting Information — On-screen instructions for the game. (DOCX) [file pone.0114976.s007.docx]
