# Supplementary material for: Gratefully Received, Gratefully Repaid: The Role of Perceived Fairness in Cooperative Interactions
Source: PLoS One. 2014 Dec 8;9(12):e114976. doi: 10.1371/journal.pone.0114976 (PMC4259482; doi:10.1371/journal.pone.0114976)
Supplement: S8 Supporting Information — Calculation of Participants’ Compensations. (DOCX) [file pone.0114976.s008.docx]

**Supporting Information 8: Calculation of Participants’ Compensations**

Of these 8 defectors the aggregate magnitude (in points, each point was worth 1p, equivalent to 0.17 cents in USD) of under-repayment (‘Expected Repayment’ minus ‘Actual Repayment’) amounted to 382 points, which was equivalent to £3.82 (US$= 6.49).

Total payoffs of P1s (N= 61) assuming NO defaulting: (12* 5.5)^a^ + (25* 5.5 – 2205/100)^b^ + [(3* 5.5)^c^ + (8*5.5)^d^ + (8* 5.5- 750/100 + 0.8* 750/100)^e^ + (5*5.5 – 360/100 + 1.2* 360/100)^f^ ] = £ 296.17
 (US$ = 503.49)

Average payoffs for P1s assuming NO defaulting: £ 296.17/ 61 = £4.86 (US$ = 8.26)

Total payoffs of P1s *after deducting P2s’ defaulting*: £ 296.17 - £3.82 = £ 292.35 (US$ = 497.0)

Average payoffs for P1s *after deducting P2s’ defaulting* : £ 292.35/ 61 = £4.79 (US$ = 8.14)

Note:
a= total payoffs for P1s who chose not to help (N=12) ,formula given by [N *(£2-standard allowance + £3.5-Bonus)];
b= total payoffs for P1s who helped unconditionally (N=25), formula given by [N *(£2-standard allowance + £3.5 Bonus) - (Points transferred /100)] ;
c= total payoffs for P1s whose offers were declined by their P2 partners (N=3) , formula given by [N *(£2-standard allowance + £3.5-Bonus)];
d = total payoffs for P1s who helped conditionally and demanded a full repayment (N=8) ,formula given by [N *(£2-standard allowance + £3.5 -Bonus)];
e= total payoffs for P1s who helped conditionally and demanded a partial, i.e. 80%, repayment (N=8) ,formula given by [N *(£2-standard allowance + £3.5 -Bonus) - (Points transferred /100) + 0.8*(Points transferred/ 100)] ;
f = total payoffs for P1s helping conditionally while demanded a repayment plus 20% surcharge (N=5), formula given by [N *(£2-standard allowance + £3.5 -Bonus) - (Points transferred /100) + 1.2* (Points transferred/ 100)]

Total payoffs of P2s (N=61) assuming NO defaulting: (12* 2)^a^ + (3*2)^b^ + (25*4 + 355/100)^c^ + (8*4 +51/100 – 651/100)^d^ + (8*4 + 100/100 – 600/100)^e^ + (5*4 + 10/100 – 432/100)^f^
= £202.33 (US$= 343.96)

Average payoffs for P2s assuming NO defaulting : £202.33/ 61 = £3.32 (US$= 5.64)

Total payoffs of P2s *inclusive of the defaulting*: £202.33 + £3.82 = £206.15 (US$= 350.46)

Average payoffs for P2s *inclusive of the defaulting*: £206.15/ 61 = £3.38 (US$= 5.75)

Note:
a= total payoffs for P2s who received no help (N=12) ,formula given by [N *(£2-allowance)];
b= total payoffs for P2s who declined their offers (N=3), formula given by [N *(£2-allowance)];
c= total payoffs for P2s who accepted unconditional offers (N=25), formula given by [N *(£2-allowance + £2-bonus for reaching the 200-point threshold) + (Total ‘extra’ points P1s transferred/ 100)];
d = total payoffs for P2s who accepted (N=8) their conditional offer which stipulated a full repayment (assuming no defaulting), formula given by [N *(£2-allowance + £2-bonus for reaching threshold)+ (Total ‘extra’ points P1s transferred/ 100)- (Total ‘obligated’repayment by recipients in points/ 100)];
e= total payoffs for P2s who accepted (N=8) their conditional offer which stipulated a partial repayment (assuming no defaulting), formula given by [N *(£2-allowance + £2-bonus for reaching threshold)+ (Total ‘extra’ points P1s transferred/ 100)- (Total ‘obligated’repayment by recipients/ 100)];
f = total payoffs for P2s who accepted (N=5) their conditional offer which stipulated a repayment plus a 20% surcharge (assuming no defaulting), formula given by [N *(£2-allowance + £2-bonus for reaching threshold)+ (Total ‘extra’ points P1s transferred/ 100)- (Total ‘obligated’repayment by recipients/ 100)];
